# Radiomics feature stability of open-source software evaluated on apparent diffusion coefficient maps in head and neck cancer

**DOI:** 10.1038/s41598-021-96600-4

**Published:** 2021-09-03

**Authors:** James C. Korte, Carlos Cardenas, Nicholas Hardcastle, Tomas Kron, Jihong Wang, Houda Bahig, Baher Elgohari, Rachel Ger, Laurence Court, Clifton D. Fuller, Sweet Ping Ng

**Affiliations:** 1 Department of Physical Science, Peter MacCallum Cancer Centre, 305 Grattan St, Melbourne, VIC 3000 Australia; 2 Department of Biomedical Engineering, University of Melbourne, Melbourne, Australia; 3 Department of Radiation Physics, University of Texas MD Anderson Cancer Center, Houston, USA; 4 Centre for Medical Radiation Physics, University of Wollongong, Wollongong, Australia; 5 Sir Peter MacCallum Department of Oncology, University of Melbourne, Melbourne, Australia; 6 Radiation Oncology Department, Centre Hospitalier de l’Université de Montréal, Montreal, Canada; 7 Department of Radiation Oncology, University of Texas MD Anderson Cancer Center, Houston, USA; 8 Clinical Oncology & Nuclear Medicine Department, Mansoura University, Mansoura, Egypt; 9 Department of Radiation Oncology, Mayo Clinic, Phoenix, AZ USA; 10 Department of Radiation Oncology, Peter MacCallum Cancer Centre, Melbourne, Australia; 11 Department of Radiation Oncology, Olivia Newton-John Cancer Wellness and Research Centre, Melbourne, Australia

**Keywords:** Cancer imaging, Image processing, Machine learning, Biomarkers

## Abstract

Radiomics is a promising technique for discovering image based biomarkers of therapy response in cancer. Reproducibility of radiomics features is a known issue that is addressed by the image biomarker standardisation initiative (IBSI), but it remains challenging to interpret previously published radiomics signatures. This study investigates the reproducibility of radiomics features calculated with two widely used radiomics software packages (IBEX, MaZda) in comparison to an IBSI compliant software package (PyRadiomics). Intensity histogram, shape and textural features were extracted from 334 diffusion weighted magnetic resonance images of 59 head and neck cancer (HNC) patients from the PREDICT-HN observational radiotherapy study. Based on name and linear correlation, PyRadiomics shares 83 features with IBEX and 49 features with MaZda, a sub-set of well correlated features are considered reproducible (IBEX: 15 features, MaZda: 18 features). We explore the impact of including non-reproducible radiomics features in a HNC radiotherapy response model. It is possible to classify equivalent patient groups using radiomic features from either software, but only when restricting the model to reliable features using a correlation threshold method. This is relevant for clinical biomarker validation trials as it provides a framework to assess the reproducibility of reported radiomic signatures from existing trials.

## Introduction

Extracting textural features from medical images provides additional information^1^ to capture changes in tumour heterogeneity that may complement existing shape based metrics^2^. Radiomics^3,4^ is the high-throughput extraction of image features from standard-of-care medical images, with the hypothesis that macroscopic image features offer insight into disease process at a molecular level^5^. Radiomics analysis has been widely adopted in oncology research, showing potential to identify magnetic resonance image (MRI) based biomarkers for clinical outcomes in head and neck cancers (HNC)^6^. The evolution of radiomics features during treatment, commonly referred to as delta-radiomics, may offer more information than a single time point to identify biomarkers during radiotherapy or chemotherapy and has been explored in HNC with CT imaging^7–10^, positron emission tomography (PET) imaging^11^ and more recently in MRI studies^12–16^.

A review of head and neck cancer studies^6^ details the investigation of MRI radiomics features for applications such as image segmentation, histopathological classification and prognostic or predictive biomarkers. Previous HNC studies explore a range of MRI sequences, from anatomical imaging such as T1 weighted, T2 weighted and short tau inversion recovery (STIR) to functional imaging such as diffusion weighted (DW-MRI) and dynamic contrast enhanced imaging (DCE-MRI). This study focuses on radiomics features calculated on apparent diffusion coefficient (ADC) maps derived from DW-MRI images. The apparent diffusion coefficient has been correlated to cellularity in many tumour types^17^ and has been linked to cell proliferation in head and neck squamous cell carcinoma^18^. During radiotherapy, early changes in ADC have been linked to treatment response outcomes for multiple tumour types, making it a potential candidate for biological image-guided adaptive radiotherapy on MRI guided radiotherapy systems^19^.

Developing a radiomics model is often considered as a series of discrete tasks each with its own challenges^5^, with the variability of each task known to impact model performance^20–22^. In MRI studies, these effects have been investigated with regard to image acquisition^23–27^, region of interest segmentation^23,28–30^, image pre-processing^28,29,31,32^, feature extraction^33–35^ and feature reduction combined with classifier training^13,36,37^. To address the known variability issues of features extracted with different software^33–35^ the image biomarker standardisation initiative (IBSI)^38^ has proposed a set of feature extraction guidelines. In head and neck cancer, MRI studies have reported feature extraction with software such as MazDa^24,39–41^, IBEX^42^ and in-house solutions based on MATLAB^37,42–49^, none of which adhear to the IBSI guidelines.

Radiomic analysis generates hundreds of features, making feature reduction a crucial step to prevent overfitting when developing a radiomics model. Validation studies^50,51^ select a small set of features based on previously reported radiomic signatures. The IBSI guidelines should mitigate known feature reproducibility issues^33–35^ in future studies, but feature uncertainty remains a problem when interpreting previously reported radiomic signatures. This study investigates the correlation between features generated with open-source radiomic software packages (IBEX^52^ and MaZda^53^) used in many published studies against an open-source tool (PyRadiomics^54^) which follows the IBSI guidelines. We then explore the impact of non-reproducible radiomics features on a HNC radiotherapy response model using through therapy ADC radiomics features. Our comparison focuses on DW-MRI of head and neck cancer but provides general confidence on which previously reported radiomics features can be reproduced with software that adheres to the IBSI guidelines.

## Results

### Variation in radiomic features

**Figure 1:**
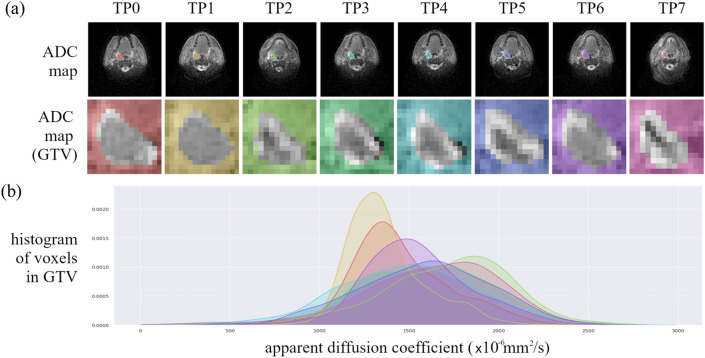
Apparent diffusion coefficient (ADC) maps of a head and neck cancer patient throughout radiotherapy from the PREDICT-HN prospective clinical trial. (**a**) ADC maps are displayed with (top row) the gross tumour volume (GTV) highlighted in colour and (middle row) cropped to the GTV to focus on the region of interest for the radiomic analysis. Change in (**b**) the ADC histogram within the GTV is from baseline (TP0), weekly throughout radiotherapy (TP1–TP6) and post-radiotherapy (TP7) with the histogram colour matched to the GTV contour colour.

**Figure 2:**
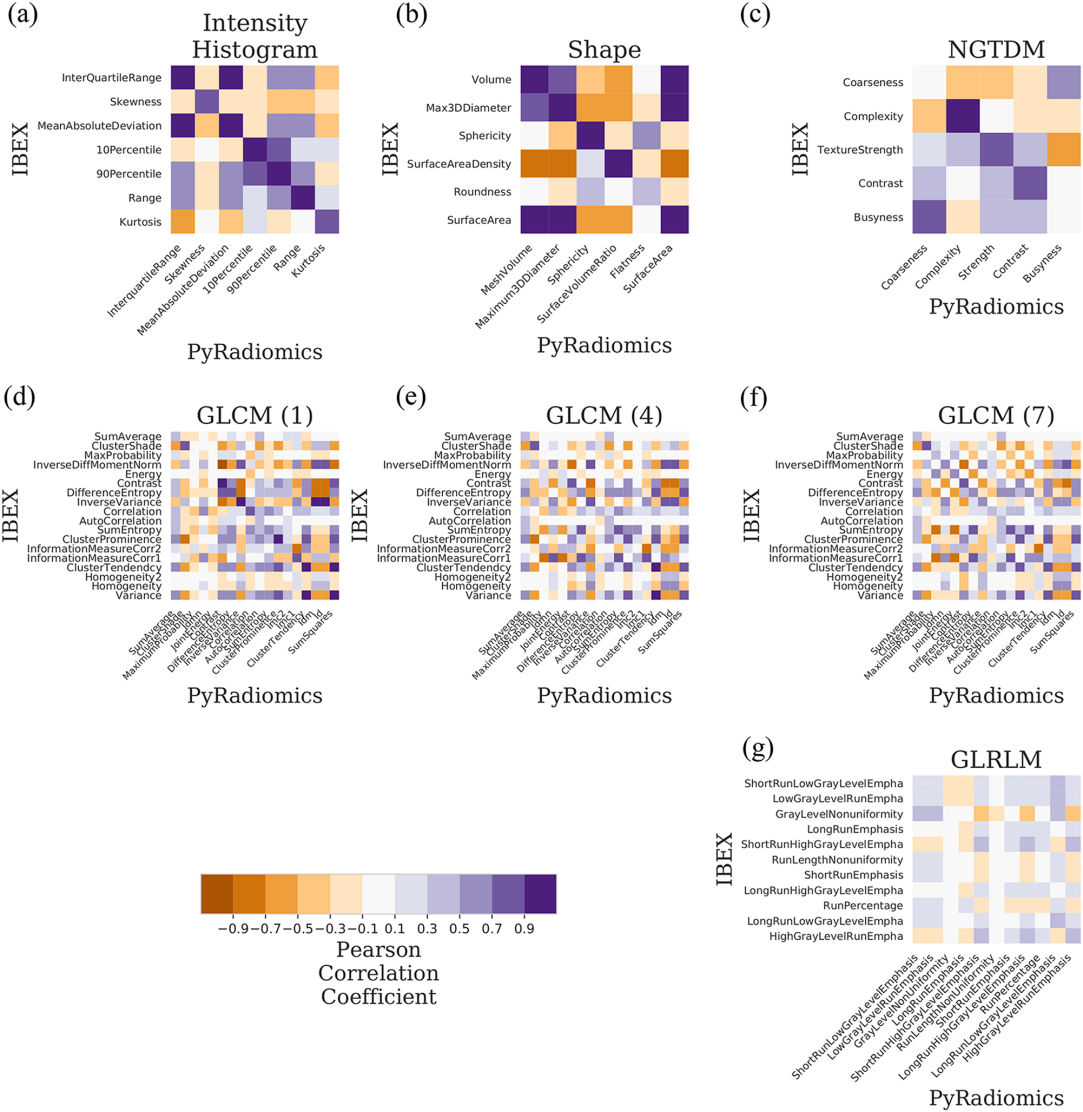
Linear correlation of apparent diffusion coefficient (ADC) radiomics features between IBEX and PyRadiomics software. Correlation matrices are grouped by feature class such as (**a**) intensity histogram (**b**) shape (**c**) NGTDM (**d**–**f**) GLCM and (**g**) GLRLM with colour representing the Pearson correlation coefficient (r). An ideal correlation matrix would have diagonal elements of highly correlated features (r = 1.0, dark purple) between software packages. A list of shared features between software packages is in Supplementary Tables 2–4.

**Figure 3:**
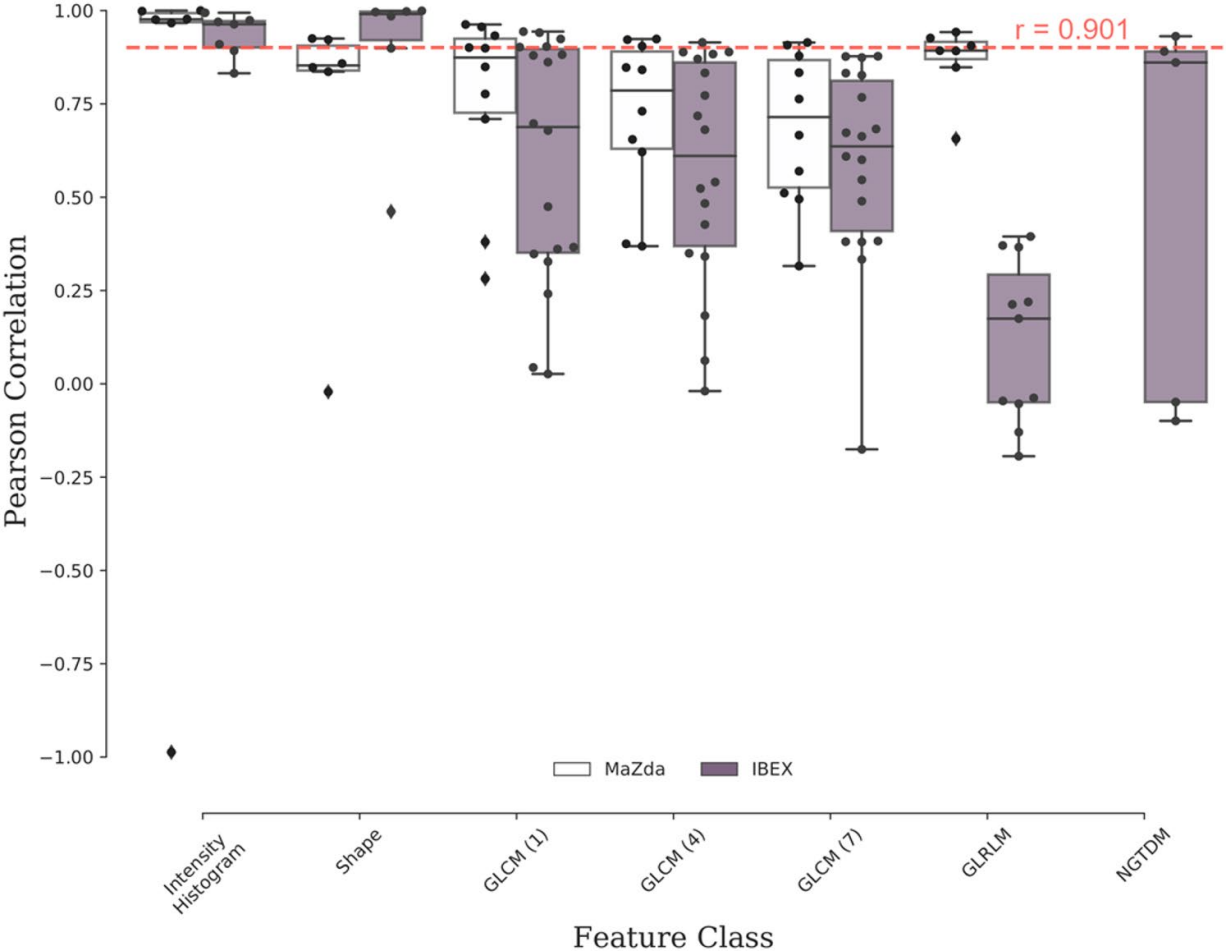
Summary of linear correlation of apparent diffusion coefficient (ADC) radiomic features between PyRadiomics and (white) MaZda and (purple) IBEX software. The reproducibility threshold (red-dashed line) is defined as greater than a Pearson correlation coefficient of 0.901. This analysis identified a sub-set of reproducible features between IBEX and PyRadiomics from intensity histogram (5/7), shape (4/6), GLCM (neighbourhood 1:4/18, 4:1/18, 7:0/18), GLRLM (0/11) and NGTDM (1/5) categories. The sub-set of reproducible features between MaZda and PyRadiomics is intensity histogram (5/6), shape (2/6), GLCM (neighbourhood 1:3/10, 3:4/10, 7:2/10), GLRLM (3/7).

Radiomics features were extracted from 334 apparent diffusion coefficient maps from the prospective PREDICT-HN study^59^(Fig. 1) with PyRadiomics, IBEX and MaZda. A total of 314 features were extracted per ADC map (PyRadiomics: 125, IBEX: 110, MaZda: 79) including intensity histogram, shape and texture features. Based on name similarity, equation similarity and linear correlation, we identified that PyRadiomics and IBEX have 83 shared features and PyRadiomics and MaZda have 49 shared features. The linear correlation of PyRadiomics and IBEX features (Fig. 2) and PyRadiomics and MaZda features (Supplementary Figure 2) shows high correlation between intensity histogram features with a range of correlation between features in the shape and texture classes. A summary of correlation between features shared with PyRadiomics (Fig. 3) shows that on average IBEX extracts more highly correlated shape features, with MaZda extracting more highly correlated first order, GLCM and GLRM features. Features with a high Pearson’s coefficient ($r>0.901$) were considered reproducible between software packages. IBEX had 15 reproducible features (intensity histogram: 5, shape: 4, GLCM(1): 4, GLCM(4): 1, NGTDM: 1) and MaZda had 18 reproducible features (intensity histogram: 5, shape: 2, GLCM(1): 3, GLCM(4): 3, GLCM(7): 2, GLRLM: 3). For full detail of the feature names and correlations see Supplementary Tables 2, 3 and 4.

The PyRadiomics feature set was composed of 18 intensity histogram, 14 shape, 24 GLCM (at three neighbourhoods), 16 GLRLM and 5 NGTDM features. The IBEX feature set had (shared/total) 7/13 intensity histogram, 6/18 shape, 18/21 GLCM (at three neighbourhoods), 11/11 GLRLM and 5/5 NGTDM features. The MaZda feature set had (shared/total) 6/14 intensity histogram, 6/22 shape, 10/12 GLCM (at three neighbourhoods) and 7/7 GLRLM features. Shared features that had dissimilar names included shape features (Flatness, Roundness), (Volume, Area), (VoxelVolume, VoxelSize), (SurfaceArea, Perimeter), (SurfaceVolumeRatio, SurfaceAreaDensity), (FerretDiameter, Maximum3DDiameter), GLCM features (SumSquares, Variance), (InverseDifference, Homogeneity1), (InverseDifferenceMoment, Homogeneity2), (JointEnergy, AngularSecondMoment), (ClusterTendancy, JointEnergy) and one GLRLM feature (RunPercentage, Fraction).

### Variation in patient modelling

**Figure 4:**
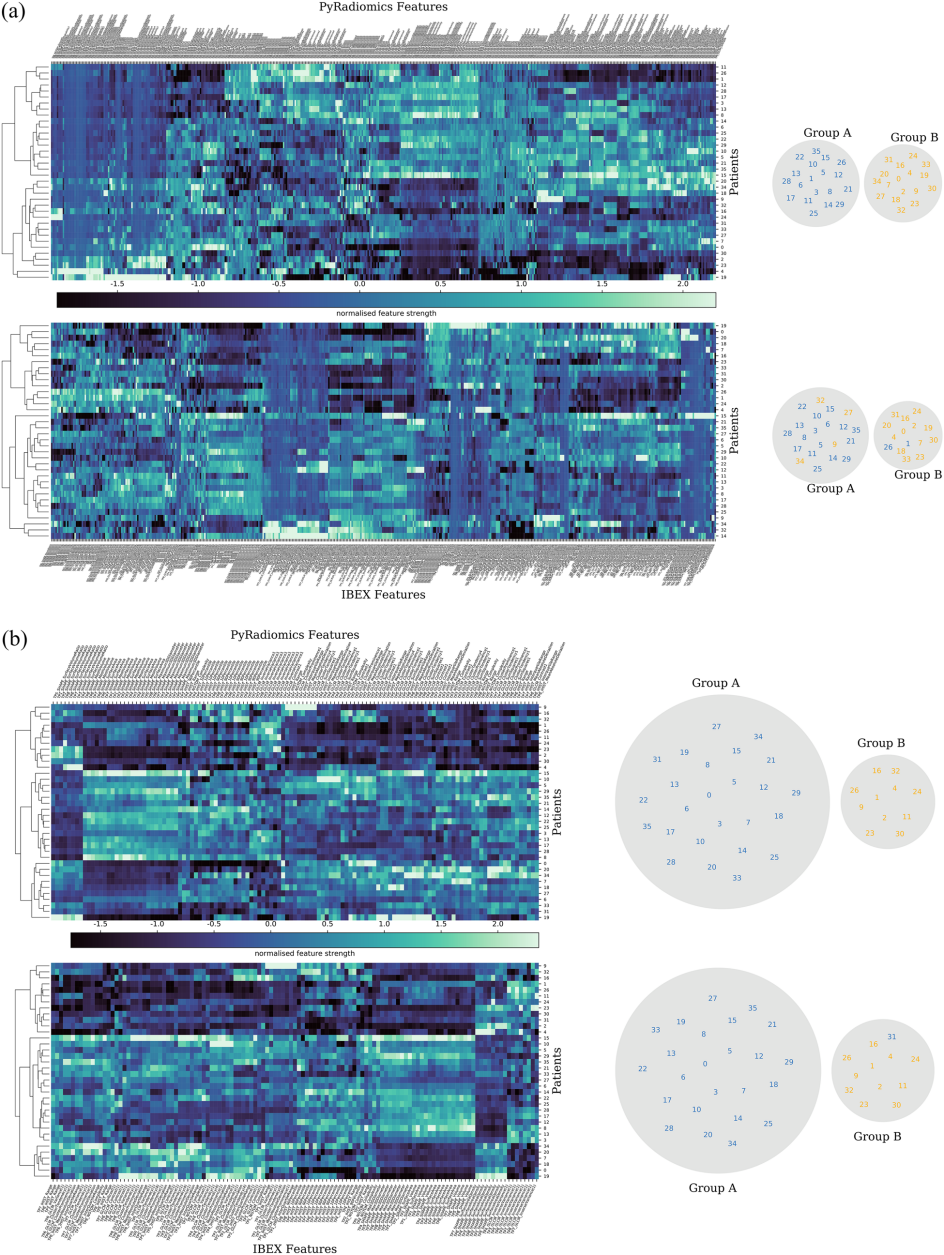
Comparison of hierarchical clustering of patients with PyRadiomics and IBEX using (**a**) all shared features and (**b**) a sub-set of reproducible features ($r> 0.901$). Unsupervised hierarchical clustering generates a (left) radiomic signature of change in apparent diffusion coefficient (ADC) features after one fraction of radiotherapy in 36 head and neck cancer patients and (right) the resulting patient groups. Clustering with (**a**) non-reproducible features creates a difference in the patient groups generated from PyRadiomics or IBEX features. Clustering with (**b**) a sub-set of reproducible features leads to almost identical patient groups generated from PyRadiomics or IBEX features.

**Figure 5:**
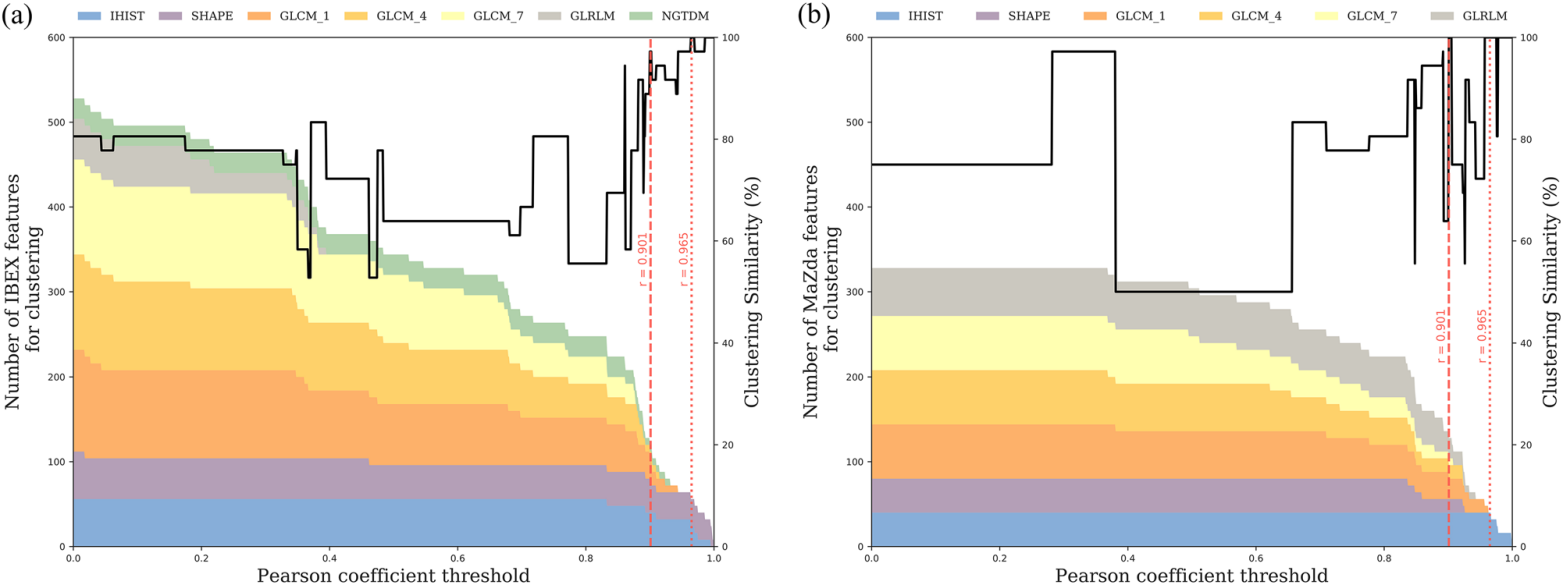
Impact of the reproducibility threshold on the number of (**a**) IBEX and (**b**) MaZda radiomics features used for clustering and the resulting clustering similarity. The number and composition of feature types is shown with the coloured area chart and shows a decrease in the number of features as the reproducibility threshold increases. The (black line) clustering similarity is relatively unchanged for a threshold up till 0.85 after which there is a general increase in accuracy for IBEX features. Two reliability thresholds are highlighted where (red dashed line) generates patient groups in IBEX with one patient classified differently and identical patient groups in MaZda and the (red dotted line) generates identical patient groups in both software.

To investigate the impact of feature variability on patient modelling, 36 patients with imaging at all eight time points were clustered into two patient groups using an unsupervised learning method. Radiomic features were calculated throughout radiotherapy on apparent diffusion maps coefficient (ADC) maps, derived from diffusion weighted MRI. Hierarchal clustering based on all shared radiomics features (Fig. 4a, Supplementary Figure 1a) resulted in different patient groups, with six patients classified differently between PyRadiomics and IBEX and fifteen patients classified differently between PyRadiomics and MaZda groups. When clustering with a reduced set of reproducible features we observed nearly identical patient groups, with one patient classified differently between PyRadiomics and IBEX radiomics features (Fig. 4b) and identical PyRadiomics and MaZda groups (Supplementary Figure 2b). The change in clustering similarity over a range of reproducibility thresholds (Fig. 5) highlights that as the reliability threshold increases the number of included radiomics features decreases, with a general trend of increasing clustering similarity for a threshold above 0.90. To cluster identical patient groups in both software a more stringent reproducibility threshold ($r>0.965$) was required and reduced the IBEX feature set to 7 features (intensity histogram: 3, GLCM(1): 4) and the MaZda feature set to 5 intensity histogram features.

## Discussion

Existing studies of variability in radiomics feature extraction^33–35^ explore a range of image modalities and feature extraction software. A study of mammograms and HNC computed tomography images^33^ also extracted IBEX and MaZda features, but compared them against one another and with two in-house software packages. One feature extraction study of HNC patients was less comparable as it analysed PET images^35^ and compared two in-house radiomics software packages. A study of HNC patients who had both CT and MRI imaging^34^ also extracted PyRadiomics features but compared them to features extracted with Moddicom^55^ and the radiomics extension to CERR^56^. Whilst that study^34^ also extracted features from MRI images of HNC patients, the features were from T2 weighted images, whereas our study is the first, to our knowledge, to investigate feature extraction stability on ADC maps from diffusion weighted MRI.

Our study observed a similar trend of feature extraction reproducibility to existing studies; intensity histogram features have high reproducibility across feature extraction software, with shape and textural features being more software package dependent. The study of mammograms and HNC CT images^33^ demonstrated high intra-class correlation (ICC) of intensity histogram features and low ICC of GLCM features, though this was partially attributed to the use of default GLCM extraction settings for each software. The study of HNC patients with both CT and MRI images^34^ showed that CT features had high Spearman correlation of intensity histogram, shape and GLCM features and lower ICC for GLRLM and grey-level size zone matrix (GLSZM) features. The T2 weighted MRI features compared in that study had high Spearman correlation for intensity histogram and shape features between PyRadiomics and CERR and only shape features highly correlated between PyRadiomics and Moddicom; the lack of correlation with intensity histogram features was attributed to Moddicom performing an image intensity correction on the T2 weighted MRI. In our study NGTDM features were extracted with IBEX and had a mixture of well correlated (1/5) and uncorrelated (4/5) features, the previously mentioned PET study^35^ also extracted NGTDM features but only a percentage of grouped textural features above an ICC threshold was reported. The similarity of our findings with those previously reported may indicate that variability in radiomics features due to the extraction software can be considered independent of the imaging modality.

Two radiomics feature reproducibility studies^34,35^ have investigated the impact of feature extraction variability on HNC model performance. The joint MRI and CT imaging study^34^ performed hierarchical clustering of patients for each feature class separately (intensity histogram, GLCM, etc.) and observed consistent clustering of clinical variables (TN category, GTV volume) for radiomics feature classes with a high reproducibility. The PET study^35^ demonstrated that models of local tumour control built on features from two different radiomics packages can stratify patients into very similar low or high risk recurrence groups, with the model features being highly correlated between the two software packages (ICC > 0.9). These studies and our results demonstrate that it is possible to generate equivalent radiomics models with different feature extraction software, but that care must be taken to ensure that features are highly correlated between the software packages. Additionally, our study extends this observation to patient classification based on radiomics features prior to treatment, at multiple time points during treatment and post-treatment.

A limitation of this and previous studies^33–35^ is the ambiguity in matching features between different software, firstly identifying shared features and secondly matching the feature extraction settings which often differ between software package. Name similarity does not guarantee equation similarity, for example the shape feature “SurfaceVolumeRatio” is calculated with a voxel based volume in IBEX and a mesh based volume in PyRadiomics. Equation similarity can be difficult to establish due to different notations, such as the formulation of the shape feature “SurfaceArea” between PyRadiomics and IBEX (Supplementary Table 6) which appear quite different but have a high correlation (0.998). We observed that equation similarity does not guarantee strong feature correlation, even with well-matched extraction settings, for a number of IBEX GLCM features such as “AutoCorrelation”, “MaximumProbability” and “JointEnergy” (Supplementary Table 7). Alternatively, minor differences in equations with non-ideally matched extraction settings showed moderate correlation, such as MaZda GLCM features “DifferenceVariance” and “InverseDifferenceMoment” (Supplementary Table 7). The naming issue can be avoided in future for a sub-set of features that have a unique identifier as defined in the IBSI guidelines^38^. To assist with consistent naming and feature extraction settings an ontology based radiomics workflow has been proposed^57^.

It is challenging to match the feature extraction settings between PyRadiomics, IBEX and MaZda. We were unable to define a reduced intensity range in MaZda for GLCM and GLRLM features, which may have negatively affected the feature correlation results, though we observed higher correlations in these feature classes from MaZda than with IBEX that had a matched intensity range and bin width. Similarly, we were unable to define identical directions for GLCM and GLRLM feature extraction across the software packages, averaging over different directions may have negatively affected our feature correlation results. A newer version of MaZda (qmazda) was selected for this study as it supports batch feature extraction, from the software documentation it was inferred that there was minimal change to the feature extraction code from earlier MaZda versions. The region of interest was calculated with a Plastimatch derived label map for PyRadiomics and MaZda and was calculated directly from the DICOM structure file (RTSTRUCT) by IBEX, which may have introduced variability in the image region used for feature extraction. We explored radiomics features calculated on the original ADC map to avoid additional variability from image filtration (Wavelet, Laplacian or Gaussian, etc.) between software implementations. Image filtration reproducibility between software using MRI images may be worth investigating as differences in wavelet filtered features from PET images have been previously reported^35^. Whilst our investigation of feature variability on patient modelling demonstrated general classification differences between software, it is challenging to quantify a clinical impact as the patient groups are not correlated with a clinical outcome. There is some evidence that unsupervised feature clustering is correlated with clinical outcomes in HNC^34^.

Magnetic resonance imaging offers insight into disease related anatomical and functional changes and is ideal for radiomics analysis as multiple MRI sequences can provide complimentary information^58^. This study reports the variability of radiomics features extracted from ADC maps, that are a relatively quantitative image, generating features not significantly influenced by scanner manufacturer or magnetic field strength^26^; radiomics features extracted from T1 and T2 weighted images are less reproducible across scanner and not recommended at this point for multicentre trials^24^. The increasing uptake of MRI for simulation and therapy in radiotherapy departments offers an unprecedented opportunity to characterise tumour response and personalise patient treatment. This study provides information on how feature extraction software can impact the reproducibility of a radiomics workflow, which we should endeavour to optimise in order to accelerate discovery through reproducible research and data sharing.

## Conclusion

This work highlights feature and model reproducibility issues due to different radiomic analysis software. We propose a correlation threshold method to select reproducible features and demonstrate that the identified features from both software generate an equivalent model. This is relevant for the selection of radiomic features in clinical biomarker validation trials as it provides a framework to assess the reproducibility of radiomic signatures from existing studies.

## Material and methods

### Study cohort

The imaging data used in this study was collected as part of the prospective PREDICT-HN study^59^ (Fig. 1). The trial imaged 59 patients with head and neck squamous cell carcinoma who were treated with curative intent radiotherapy, patient details are summarised in Table 1. Trial participants were imaged prior to radiotherapy, weekly during radiotherapy (following fraction 5, 10, 15, 20, 25 and 30) and two to three months post-radiotherapy. Imaging data was acquired for all patients prior to radiotherapy (n = 59), with a lower number of images acquired during radiotherapy over the first three weeks (n = 40), at weeks four and five (n = 39), at week six (n = 38) and post-radiotherapy (n = 39).

**Table 1:** Patient characteristics.

	Correlation cohort (n = 59)	Cluster cohort (n = 36)
**Sex**		
Male	50	32
Female	9	4
Age (median, range)	59 (41–81)	60 (41–81)
**Primary site**		
Oropharynx	39	26
Larynx	7	2
Nasopharynx	9	4
Nasal cavity	1	1
Unknown primary	3	3
**T stage**		
T0	3	3
T1	8	5
T2	20	12
T3	12	7
T4	16	9
**N stage**		
N0	11	5
N1	9	5
N2	38	26
N3	1	0
Photon	42	24
Proton	17	12
Radiation dose (cGy, median, range)	6996 (6600–7000)	
Number of fractions	33 (33–35)	

#### Ethics approval and consent to participate

The PREDICT-HN study^59^ was conducted in accordance with the Declaration of Helsinki and was approved by the Institutional Review Board at the University of Texas MD Anderson Cancer Center. The study is registered on clinicaltrials.gov with registration number NCT03491176, date of registration 09/04/2018 (retrospectively registered), date of enrolment of the first participant 30/05/2017. All study participants were 18 years and older, informed consent was obtained from all participants.

### Imaging protocol

MRI imaging was performed on a Siemens 1.5 T Aera scanner to acquire both anatomical and functional images. The gross tumour volume (GTV) was contoured on pre-treatment T2 weighted turbo-spin-echo (T2w-TSE) images (voxel size = 0.5 mm, FOV = 256 × 256 mm, axial slices = 12, slice thickness = 2 mm, TE = 80 ms, TR = 4800 ms, FA = 90°, ETL = 15, pixel bandwidth = 300 Hz) by a radiation oncologist. Throughout treatment, the pre-treatment contours were rigidly registered to the through treatment images and manually adjusted to anatomical boundaries, then propagated onto the apparent diffusion coefficient (ADC) maps and visually verified. Diffusion weighted images (DWI) were acquired with the BLADE^60,61^ sequence (voxel size = 2 mm, FOV = 256 × 256 mm, axial slices = 25, slice thickness = 4 mm, TE = 50 ms, TR = 5400 ms, FA = 90°, b = 0,800 s/mm2, ETL = 15, pixel bandwidth = 1220 Hz). Apparent diffusion coefficient maps were calculated from the DWI images using the default mono-exponential model.

### Feature extraction

Features were extracted from all the ADC maps (n = 334) using PyRadiomics (version 2.1.0), IBEX (version 1.0 Beta) and MaZda (qmazda 19.02). All available intensity histogram (IHIST), shape, grey-level co-occurrence matrix (GLCM^62^), grey-level run length matrix (GLRLM^63^) and neighbourhood grey-tone difference matrix (NGTDM^64^) features were calculated on the original ADC map only; the reproducibility of image filtration prior to feature extraction was considered outside the scope of this study. The region of interest for feature extraction was calculated directly from the DICOM radiotherapy contours (RTSTRUCT) with IBEX and an intermediate step to convert the DICOM contours to a binary label map with Plastimatch (version 1.7.3)^65^ was required for PyRadiomics and MaZda. The feature extraction settings (Supplementary Table 1) were set as the default IBEX settings and matched as closely as possible between the three software packages, based on available documentation. ADC maps were discretised (256 bins, bin width = 16) prior to calculation of IHIST and NGTDM features and with a reduced data range (100 bins, bin width = 21) for GLCM and GLRLM features. GLCM features were calculated at a series of neighbourhoods (1, 4, 7), asymmetric features were calculated with PyRadiomics and IBEX, symmetric features were calculated with MaZda to compensate for a reduced range of direction angles. Symmetric NGTDM features were calculated with a neighbourhood of three. Texture features (GLCM, GLRLM, NGTDM) were calculated in the axial plane over the three dimensional region of interest,an example of this is the NGTDM matrices being constructed with a 2D neighbourhood rather than a 3D neighbourhood. We calculated the average of all GLCM and GLRLM feature directions as PyRadiomics does not report or allow the specification of features for individual directions.

### Variation in radiomic features

To determine the relationship between features generated with the established radiomics software (IBEX/MaZda) and the IBSI compliant software (PyRadiomics) we performed a linear regression analysis on radiomics features extracted from ADC maps of all patients at all time points. A linear fit was calculated between PyRadiomics and IBEX or MaZda for every feature in a given feature class (IHIST, SHAPE, GLCM, GLRLM, NGTDM). Features with an invalid value (i.e. infinity due to division by zero) were excluded during the linear regression. A list of shared radiomics features was collated by identifying features extracted by both PyRadiomics and the alternative software package, and was based primarily on name and equation similarity. A small number of features that showed a high linear correlation but had dissimilar names, for example ‘SurfaceArea’ and ‘Perimeter’, were also included. The list of shared features, feature correlations and the number of images used per feature correlation can be found in Supplementary Tables 2, 3 and 4. Full details of the equation comparison are in Supplementary Tables 5–9.

### Variation in patient modelling

To demonstrate the potential impact of incorporating non-reproducible features in a radiomics model we used unsupervised learning to identify two groups of patients, based on radiomic features at pre-treatment, throughout radiotherapy and post-radiotherapy. Separate radiomics models were generated based on PyRadiomics and IBEX/MaZda features, first using all features and then with the sub-set of reproducible features. Reproducible features were selected as those with a high Pearson’s correlation coefficient ($r> 0.901$) as calculated for the analysis of variation in radiomics features, the correlation threshold was determined as per the sensitivity analysis described below. Features that contained an invalid number (i.e. infinity) for any patient in the modelling cohort were excluded. Patients (n = 36) with image data for all time points were grouped with SciPy^66,67^ using Ward's minimum variance clustering method^68^ on scaled radiomics features (z-score standardisation) with an automatic minimum clustering threshold to generate no more than two clusters.

To test the sensitivity of clustering to the selected correlation threshold, we performed the clustering over a range of thresholds ($r>0.0$ to $r>0.999$, with an increment of 0.001) and measured the clustering similarity as the percentage of patients clustered by IBEX or MaZda into the same groups as PyRadiomics. We define clustering similarity as,

$$ Similarity_{i} \;\left( \% \right) = \frac{{ \left| {A_{PyRad} \cap A_{i} } \right| + |B_{PyRad} \cap B_{i} |}}{{\left| {A_{PyRad} \cup B_{PyRad} } \right|}}100, $$

where ${A}_{PyRad}$ and ${B}_{PyRad}$ are patient groups from clustering with PyRadiomics features, ${A}_{i}$ and ${B}_{i}$ are patient groups from clustering with either IBEX or MaZda features, set intersection is denoted with $\cap$, set union is denoted with $\cup$ and the number of patients in a set is denoted with $|A|$. Due to the possibility that unsupervised clustering can return similar groups but in a different order, the similarity metric was calculated as,

$$Similarity_{i} \;\left( \% \right) = max \left( {\frac{{ \left| {A_{PyRad} \cap A_{i} } \right| + |B_{PyRad} \cap B_{i} |}}{{\left| {A_{PyRad} \cup B_{PyRad} } \right|}} , \frac{{ \left| {A_{PyRad} \cap B_{i} } \right| + |B_{PyRad} \cap A_{i} |}}{{\left| {A_{PyRad} \cup B_{PyRad} } \right|}}} \right) 100.$$

## Supplementary Information

Supplementary Information.
