# Author Correction: Radiomics feature stability of open-source software evaluated on apparent diffusion coefficient maps in head and neck cancer

**DOI:** 10.1038/s41598-021-98445-3

**Published:** 2021-09-17

**Authors:** James C. Korte, Carlos Cardenas, Nicholas Hardcastle, Tomas Kron, Jihong Wang, Houda Bahig, Baher Elgohari, Rachel Ger, Laurence Court, Clifton D. Fuller, Sweet Ping Ng

**Affiliations:** 1 Department of Physical Science, Peter MacCallum Cancer Centre, 305 Grattan St, Melbourne, VIC 3000 Australia; 2 Department of Biomedical Engineering, University of Melbourne, Melbourne, Australia; 3 Department of Radiation Physics, University of Texas MD Anderson Cancer Center, Houston, USA; 4 Centre for Medical Radiation Physics, University of Wollongong, Wollongong, Australia; 5 Sir Peter MacCallum Department of Oncology, University of Melbourne, Melbourne, Australia; 6 Radiation Oncology Department, Centre Hospitalier de l’Université de Montréal, Montreal, Canada; 7 Department of Radiation Oncology, University of Texas MD Anderson Cancer Center, Houston, USA; 8 Clinical Oncology & Nuclear Medicine Department, Mansoura University, Mansoura, Egypt; 9 Department of Radiation Oncology, Mayo Clinic, Phoenix, AZ USA; 10 Department of Radiation Oncology, Peter MacCallum Cancer Centre, Melbourne, Australia; 11 Department of Radiation Oncology, Olivia Newton-John Cancer Wellness and Research Centre, Melbourne, Australia

Correction to: *Scientific Reports* 10.1038/s41598-021-96600-4, published online 03 September 2021

The original version of this Article contained an error in the order of the Figures. Figures 1, 2, 3, 4 and 5 were published as Figures 5, 1, 2, 3, and 4 respectively.

The original Figures 1, 2, 3, 4 and 5 and accompanying legends appear below.

**Figure 1:**
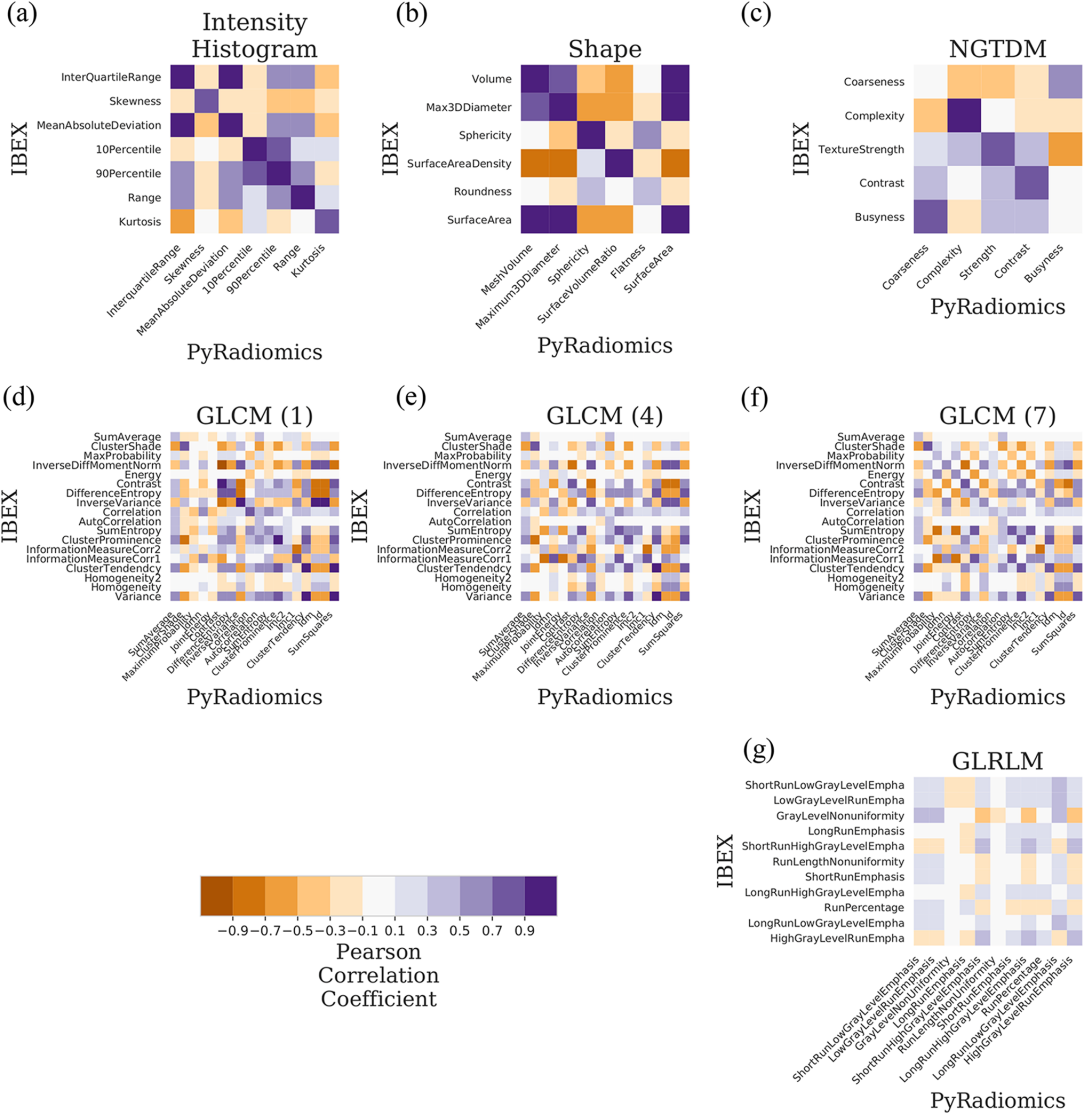
Apparent diffusion coefficient (ADC) maps of a head and neck cancer patient throughout radiotherapy from the PREDICT-HN prospective clinical trial. (**a**) ADC maps are displayed with (top row) the gross tumour volume (GTV) highlighted in colour and (middle row) cropped to the GTV to focus on the region of interest for the radiomic analysis. Change in (**b**) the ADC histogram within the GTV is from baseline (TP0), weekly throughout radiotherapy (TP1–TP6) and post-radiotherapy (TP7) with the histogram colour matched to the GTV contour colour.

**Figure 2:**
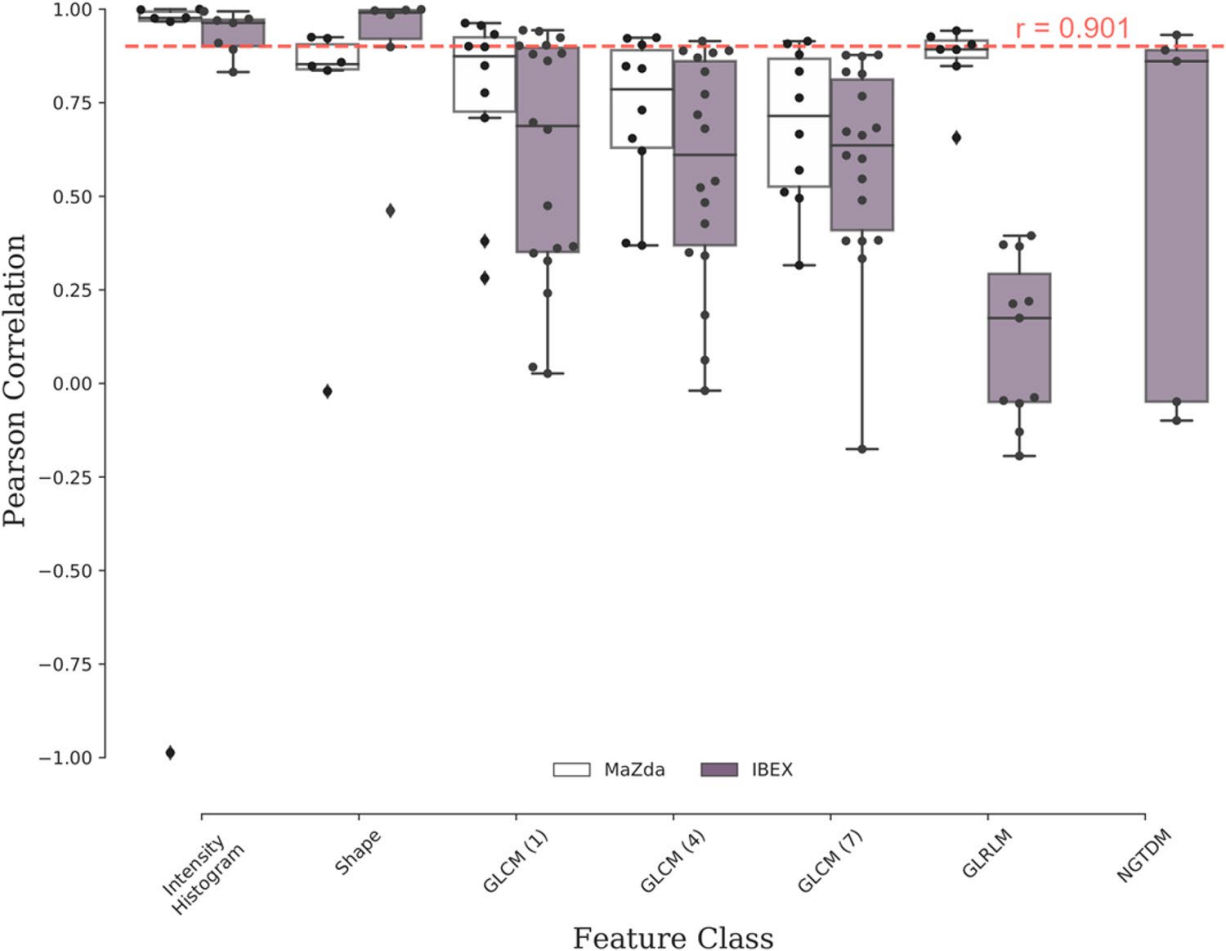
Linear correlation of apparent diffusion coefficient (ADC) radiomics features between IBEX and PyRadiomics software. Correlation matrices are grouped by feature class such as (**a**) intensity histogram (**b**) shape (**c**) NGTDM (**d**–**f**) GLCM and (**g**) GLRLM with colour representing the Pearson correlation coefficient (r). An ideal correlation matrix would have diagonal elements of highly correlated features (r = 1.0, dark purple) between software packages. A list of shared features between software packages is in Supplementary Tables 2–4.

**Figure 3:**
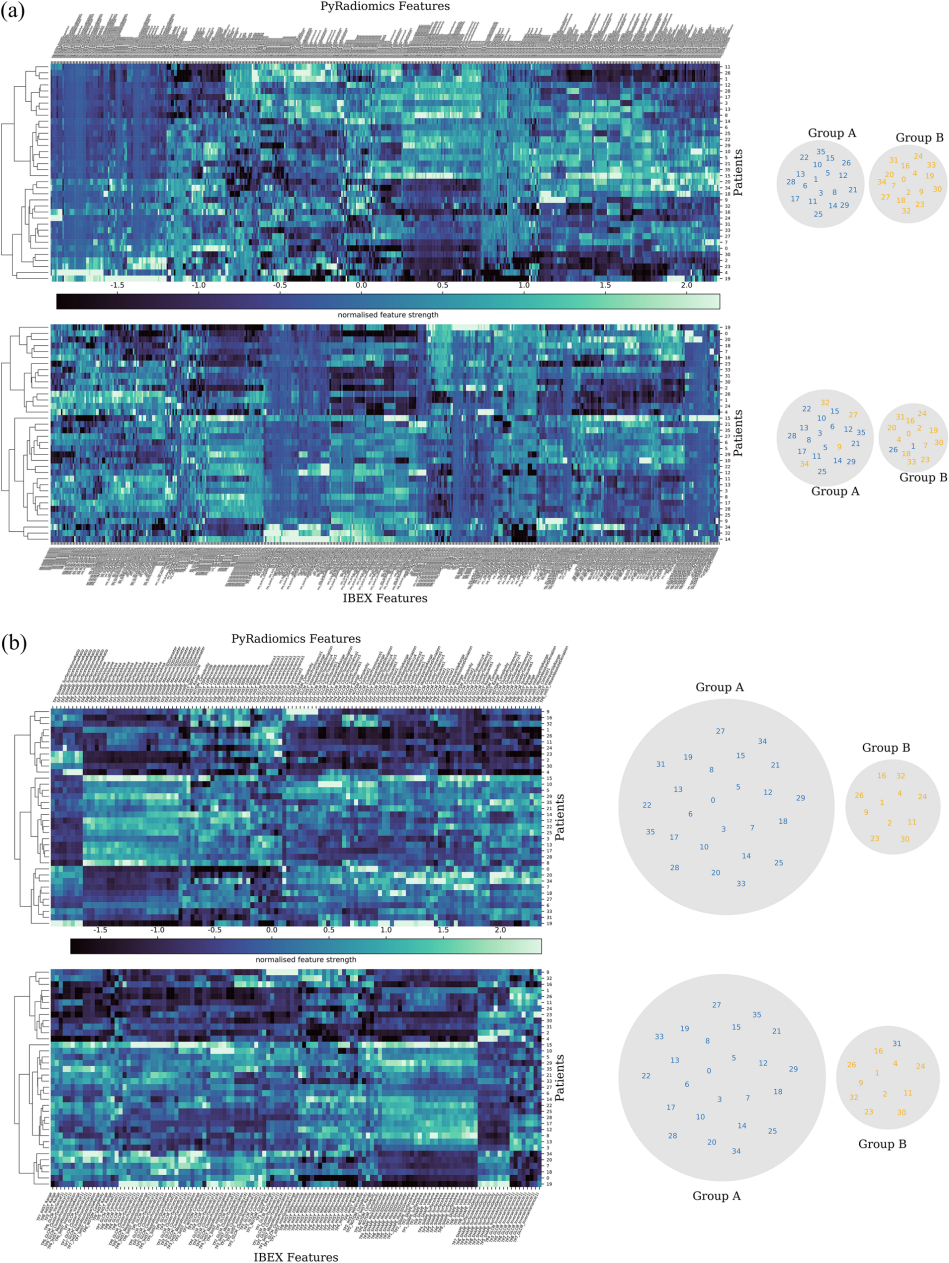
Summary of linear correlation of apparent diffusion coefficient (ADC) radiomic features between PyRadiomics and (white) MaZda and (purple) IBEX software. The reproducibility threshold (red-dashed line) is defined as greater than a Pearson correlation coefficient of 0.901. This analysis identified a sub-set of reproducible features between IBEX and PyRadiomics from intensity histogram (5/7), shape (4/6), GLCM (neighbourhood 1:4/18, 4:1/18, 7:0/18), GLRLM (0/11) and NGTDM (1/5) categories. The sub-set of reproducible features between MaZda and PyRadiomics is intensity histogram (5/6), shape (2/6), GLCM (neighbourhood 1:3/10, 3:4/10, 7:2/10), GLRLM (3/7).

**Figure 4:**
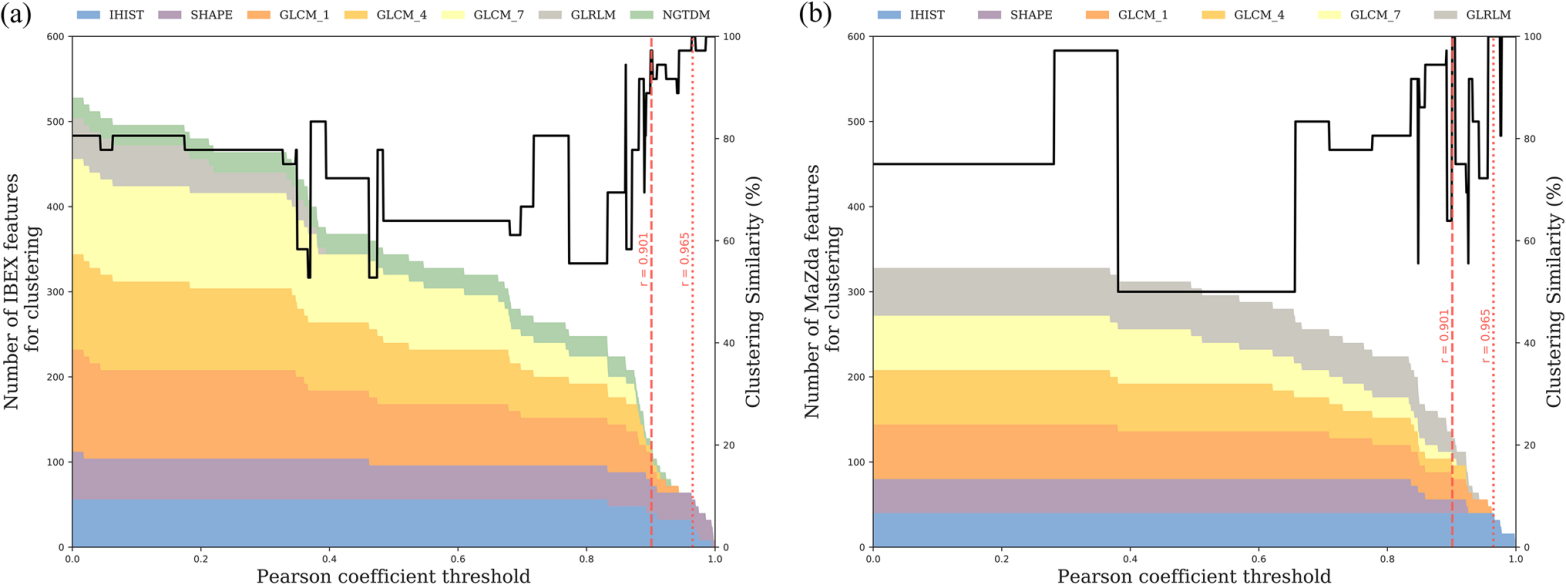
Comparison of hierarchical clustering of patients with PyRadiomics and IBEX using (**a**) all shared features and (**b**) a sub-set of reproducible features ($r> 0.901$). Unsupervised hierarchical clustering generates a (left) radiomic signature of change in apparent diffusion coefficient (ADC) features after one fraction of radiotherapy in 36 head and neck cancer patients and (right) the resulting patient groups. Clustering with (**a**) non-reproducible features creates a difference in the patient groups generated from PyRadiomics or IBEX features. Clustering with (**b**) a sub-set of reproducible features leads to almost identical patient groups generated from PyRadiomics or IBEX features.

**Figure 5:**
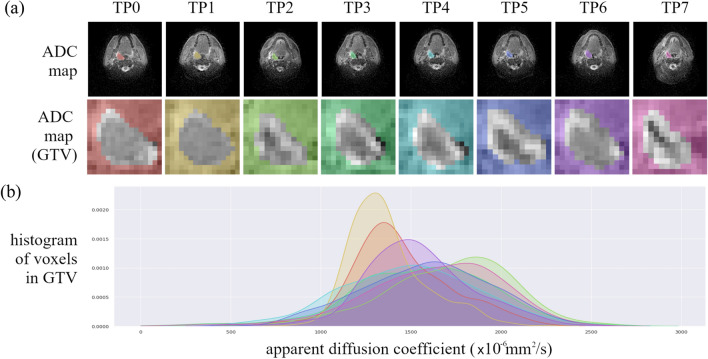
Impact of the reproducibility threshold on the number of (**a**) IBEX and (**b**) MaZda radiomics features used for clustering and the resulting clustering similarity. The number and composition of feature types is shown with the coloured area chart and shows a decrease in the number of features as the reproducibility threshold increases. The (black line) clustering similarity is relatively unchanged for a threshold up till 0.85 after which there is a general increase in accuracy for IBEX features. Two reliability thresholds are highlighted where (red dashed line) generates patient groups in IBEX with one patient classified differently and identical patient groups in MaZda and the (red dotted line) generates identical patient groups in both software.

The original Article has been corrected.

