# Supplementary material for: Radiomics feature stability of open-source software evaluated on apparent diffusion coefficient maps in head and neck cancer
Source: Sci Rep. 2021 Sep 3;11:17633. doi: 10.1038/s41598-021-96600-4 (PMC8417253; doi:10.1038/s41598-021-96600-4)
Supplement: Supplementary file 1 — Supplementary Information. [file 41598_2021_96600_MOESM1_ESM.pdf]

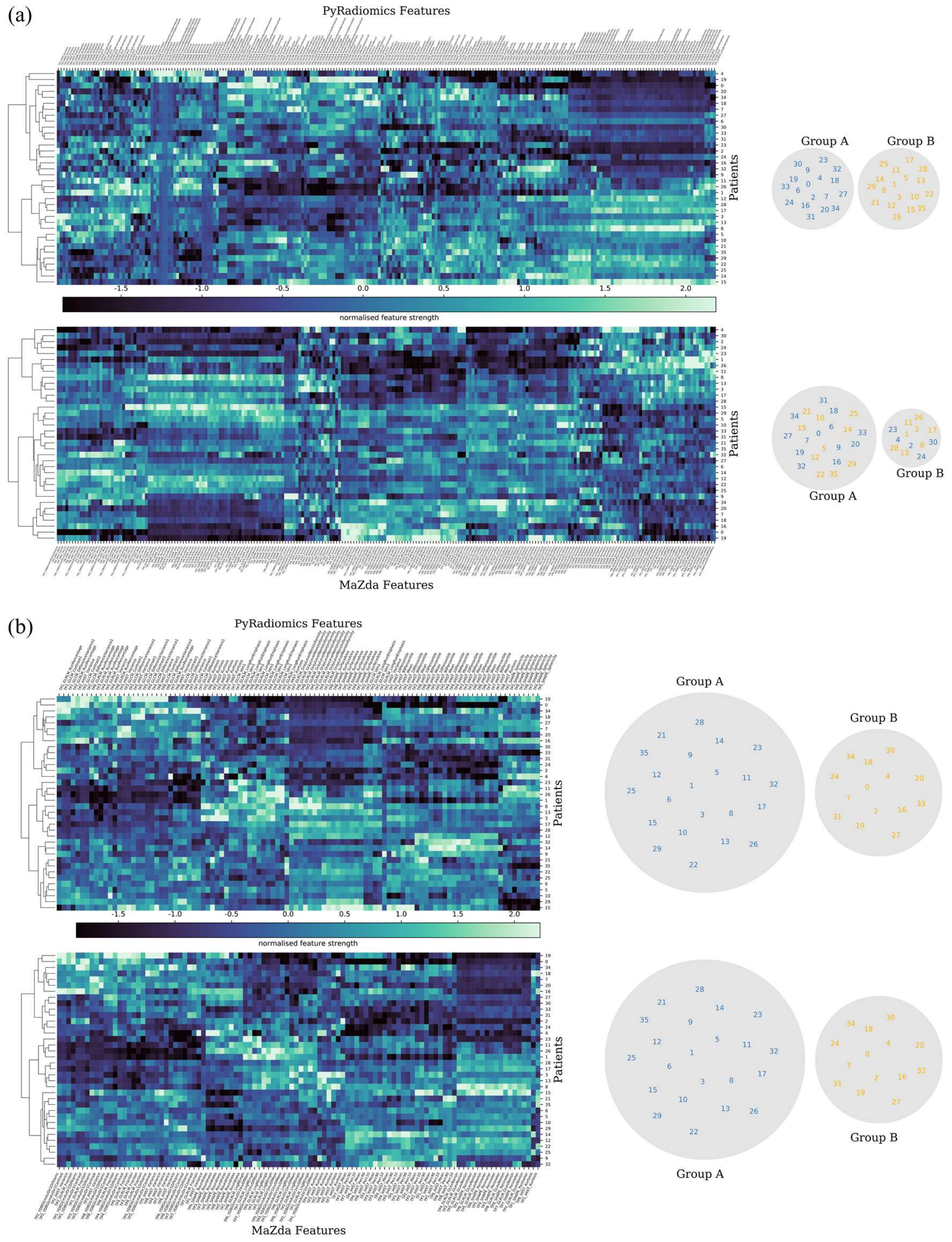

Supplementary Figure 1: Comparison of hierarchical clustering of patients with PyRadiomics and MaZda ($r > 0.901$) using (a) all shared features and (b) a sub-set of reproducible features. Unsupervised hierarchical clustering generates a (left) radiomic signature of change in apparent diffusion coefficient (ADC) features after one fraction of radiotherapy in 36 head and neck cancer patients and (right) the resulting patient groups. Clustering with (a) non-reproducible features creates a difference in the patient groups generated from PyRadiomics or MaZda features. Clustering with (b) a sub-set of reproducible features leads to identical patient groups generated from PyRadiomics or MaZda features.

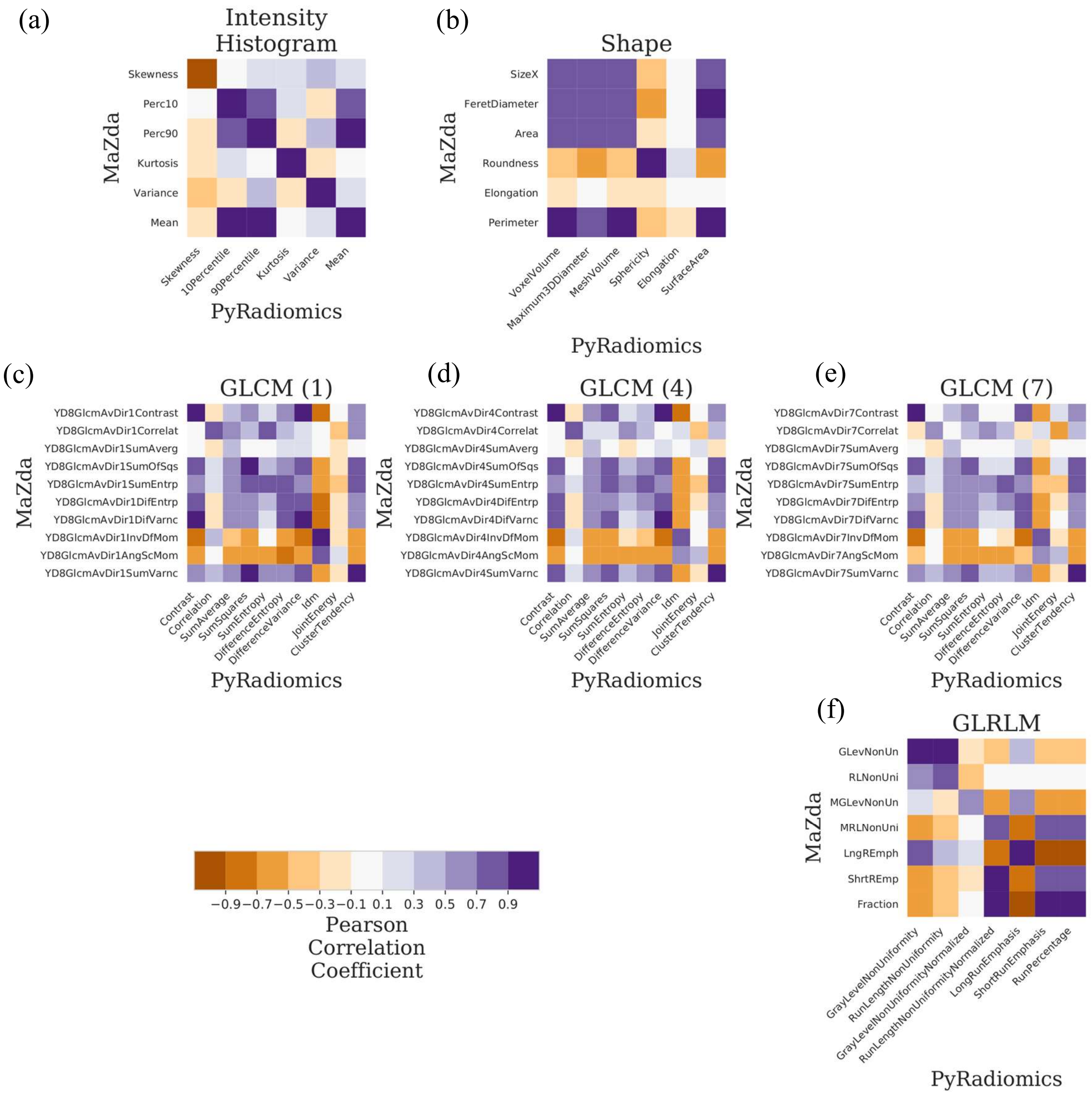

Supplementary Figure 2: Correlation maps of radiomics features calculated in MaZda and PyRadiomics. Correlation matrices are grouped by feature class such as (a) intensity histogram (b) shape (c-e) GLCM and (f) GLRLM with colour representing the Pearson correlation coefficient (r). An ideal correlation matrix would have diagonal elements of highly correlated features (r=1.0, dark purple) between software packages. A list of shared features between software packages is in Supplementary Table 2-4

	PyRadiomics (2.1.0)	IBEX (V1.0 beta)	MaZda (qmazda 19.02)
**Intensity Histogram**	Discretised (256 bins, bin width 16)<br>18 features	Discretised (256 bins, bin width 16)<br>13 features	Discretised (256 bins, bin width 16)<br>14 features
**Shape**	14 features	18 features	22 features
**Grey-level co-occurrence (GLCM)**	Grey level limits:<br>[0, 2100]<br>Discretised (100 bins, bin width 21)<br>Neighbourhoods: 1, 4, 7<br>Calculated on axial slices<br>Non-symmetric<br>Directions per neighbourhood:<br>(n=1): 0, 45, 90, 135, 180, 225, 270, 315<br>(n=4): 0, 11.25, 22.5, …, 348.75<br>(n=7): 0, 6.43, 12.86, …, 353.57<br><br>24 features	Grey level limits:<br>[0, 2100]<br>Discretised (100 bins, bin width 21)<br>Neighbourhoods: 1, 4, 7<br>Calculated on axial slices<br>Non-symmetric<br>Directions (degrees):<br>0, 45, 90, 135, 180, 225, 270, 315<br><br>21 features	Grey level limits:<br>[0, 4096]<br>Discretised (256 bins, bin width 16)<br>Neighbourhoods: 1, 4, 7<br>Calculated on axial slices<br>Symmetric<br>Directions (degrees):<br>0, 45, 90, 135<br><br>12 features
**Grey-level run length (GLRLM)**	Grey level limits:<br>[0, 2100]<br>Discretised (100 bins, bin width 21)<br>Calculated on axial slices<br>Directions not documented<br><br>16 features	Grey level limits:<br>[0, 2100]<br>Discretised (100 bins, bin width 21)<br>Calculated on axial slices<br>Direction: 0, 90 degrees<br><br>11 features	Grey level limits:<br>[0, 4096]<br>Discretised (256 bins, bin width 16)<br>Calculated on axial slices<br>Direction: 0, 90 degrees<br><br>7 features
**Neighbourhood grey tone difference (NGTDM)**	Discretised (256 bins, bin width 16)<br>Neighbourhood 3<br>Calculated on axial slices<br><br>5 features	Discretised (256 bins, bin width 16)<br>Neighbourhood 3<br>Calculated on axial slices<br><br>5 features	-

Supplementary Table 1: Feature extraction software settings for PyRadiomics, IBEX and MaZda.

Feature Class	Feature Name				Pearsons Coefficient		Number of Images	
	Shared	*PyRadiomics*	*MaZda*	*IBEX*	*MaZda*	*IBEX*	*MaZda*	*IBEX*
Intensity Histogram	Skewness	firstorder_Skewness	YD8HistSkewness	F1-IntensityHistogram_Skewness	-0.987	0.892	334	334
	10Percentile	firstorder_10Percentile	**YD8HistPerc10***	**F1-IntensityHistogram_10Percentile***	**0.967**	**0.974**	334	334
	90Percentile	firstorder_90Percentile	**YD8HistPerc90***	**F1-IntensityHistogram_90Percentile***	**0.976**	**0.994**	334	334
	Kurtosis	firstorder_Kurtosis	**YD8HistKurtosis***	F1-IntensityHistogram_Kurtosis	**0.977**	0.832	334	334
	Variance	firstorder_Variance	**YD8HistVariance***		**0.999**		334	
	Mean	firstorder_Mean	**YD8HistMean***		**1**		334	
	InterquartileRange	firstorder_InterquartileRange		**F1-IntensityHistogram_InterQuartileRange**		**0.963**		334
	MeanAbsDev	firstorder_MeanAbsoluteDeviation		**F1-IntensityHistogram_MeanAbsoluteDeviation***		**0.97**		334
	Range	firstorder_Range		**F1-IntensityHistogram_Range**		**0.91**		334
		firstorder_Uniformity						
		firstorder_Median						
		firstorder_Energy						
		firstorder_RobustMeanAbsoluteDeviation						
		firstorder_TotalEnergy						
		firstorder_Maximum						
		firstorder_RootMeanSquared						
		firstorder_Minimum						
		firstorder_Entropy						
			YD8HistArea	F1-IntensityHistogram_MedianAbsoluteDeviation				
			YD8HistPerc01	F1-IntensityHistogram_10PercentileArea				
			YD8HistPerc50	F1-IntensityHistogram_90PercentileArea				
			YD8HistPerc99	F1-IntensityHistogram_ 0.25Quantile				
			YD8HistMaxm01	F1-IntensityHistogram_ 0.5Quantile				
			YD8HistMaxm10	F1-IntensityHistogram_ 0.75Quantile				
			YD8HistDomn10					
			YD8HistArea					
			YD8HistDomn01					
Shape	VoxelVolume	shape_VoxelVolume	MorltkSizeX		0.836		334	
	Max3DDiameter	shape_Maximum3DDiameter	MorltkFeretDiameter	**F2-Shape_Max3DDiameter***	0.858	**0.996**	334	334
	MeshVolume	shape_MeshVolume	MorltkArea	**F2-Shape_Volume***	0.848	**0.999**	334	334
	Sphericity	shape_Sphericity	**MorltkRoundness**	F2-Shape_Sphericity	**0.925**	0.899	334	334
	Elongation	shape_Elongation	MorltkElongation		-0.021		334	
	SurfaceArea	shape_SurfaceArea	**MorltkPerimeter**	**F2-Shape_SurfaceArea***	**0.922**	**0.998**	334	334
	SurfaceVolumeRatio	shape_SurfaceVolumeRatio		**F2-Shape_SurfaceAreaDensity***		**0.985**		334
	Flatness	shape_Flatness		F2-Shape_Roundness		0.462		334
		shape_MajorAxisLength						
		shape_LeastAxisLength						
		shape_Maximum2DDiameterSlice						
		shape_MinorAxisLength						
		shape_Maximum2DDiameterColumn						
		shape_Maximum2DDiameterRow						
			MorltkFullyConnectedFalse	F2-Shape_VoxelSize				
			MorltkFullyConnectedTrue	F2-Shape_SphericalDisproportion				
			MorltkCentroidX	F2-Shape_Compactness1				
			MorltkCentroidY	F2-Shape_Compactness2				
			MorltkCentroidZ	F2-Shape_Convex				
			MorltkPrincipalMomentX	F2-Shape_ConvexHullVolume				
			MorltkPrincipalMomentY	F2-Shape_ConvexHullVolume3D				
			MorltkPrincipalMomentZ	F2-Shape_Mass				
			MorltkTilt	F2-Shape_MeanBreadth				
			MorltkEquivalentSphericalRadius	F2-Shape_NumberOfObjects				
			MorltkEquivalentEllipsoidDiameter[illegible]	F2-Shape_NumberOfVoxel				
			MorltkEquivalentEllipsoidDiameter[illegible]	F2-Shape_Orientation				
			MorltkEquivalentEllipsoidDiameterZ					
			MorltkEquivalentSphericalPerimeter					
			MorltkSizeZ					
			MorltkSizeY					

Supplementary Table 2: Features shared between PyRadiomics, MaZda and IBEX and their linear correlation on a set of HNC ADC maps. Features highlighted with (bold text) have a linear correlation coefficient, $r > 0.901$, (bold text with an asterisk) a linear correlation, $r > 0.965$ and (grey text) unmatched features.

Feature Class	Feature Name				Pearsons Coefficient		Number of Images	
	Shared	*PyRadiomics*	*MaZda*	*IBEX*	*MaZda*	*IBEX*	*MaZda*	*IBEX*
	Contrast	glcm_Contrast1	**YD8GlcmAvDir1Contrast**	**F4-GrayLevelCooccurenceMatrix25_-333-1Contrast**	**0.963**	**0.924**	334	334
	Correlation	glcm_Correlation1	YD8GlcmAvDir1Correlat	F4-GrayLevelCooccurenceMatrix25_-333-1Correlation	0.777	0.862	334	334
	SumAverage	glcm_SumAverage1	YD8GlcmAvDir1SumAverg	F4-GrayLevelCooccurenceMatrix25_-333-1SumAverage	0.38	0.361	334	334
	SumSquares	glcm_SumSquares1	YD8GlcmAvDir1SumOfSqs	**F4-GrayLevelCooccurenceMatrix25_-333-1Variance**	0.901	**0.941**	334	334
	SumEntropy	glcm_SumEntropy1	YD8GlcmAvDir1SumEntrp	F4-GrayLevelCooccurenceMatrix25_-333-1SumEntropy	0.709	0.679	334	334
	DifferenceEntropy	glcm_DifferenceEntropy1	YD8GlcmAvDir1DifEntrp	F4-GrayLevelCooccurenceMatrix25_-333-1DifferenceEntropy	0.849	0.697	334	334
	DifferenceVariance	glcm_DifferenceVariance1	**YD8GlcmAvDir1DifVarnc**		**0.956**		334	
	InverseDifferenceMoment	glcm_Idm1	**YD8GlcmAvDir1InvDfMom**	F4-GrayLevelCooccurenceMatrix25_-333-1Homogeneity2	**0.933**	0.241	334	334
	JointEnergy	glcm_JointEnergy1	YD8GlcmAvDir1AngScMom	F4-GrayLevelCooccurenceMatrix25_-333-1Energy	0.282	0.044	334	334
	ClusterTendency	glcm_ClusterTendency1	YD8GlcmAvDir1SumVarnc	**F4-GrayLevelCooccurenceMatrix25_-333-1ClusterTendendcy**	0.899	**0.944**	334	334
Grey-level co-occurrence (GLCM) Neighbourhood = 1	ClusterShade	glcm_ClusterShade1		F4-GrayLevelCooccurenceMatrix25_-333-1ClusterShade		0.882		334
	MaxProbability	glcm_MaximumProbability1		F4-GrayLevelCooccurenceMatrix25_-333-1MaxProbability		0.027		334
	Idmn	glcm_Idmn1		F4-GrayLevelCooccurenceMatrix25_-333-1InverseDiffMomentNorm		0.348		334
	InverseVariance	glcm_InverseVariance1		F4-GrayLevelCooccurenceMatrix25_-333-1InverseVariance		**0.903**		334
	AutoCorrelation	glcm_Autocorrelation1		F4-GrayLevelCooccurenceMatrix25_-333-1AutoCorrelation		0.366		334
	ClusterProminence	glcm_ClusterProminence1		**F4-GrayLevelCooccurenceMatrix25_-333-1ClusterProminence**		**0.902**		334
	Imc2	glcm_Imc21		F4-GrayLevelCooccurenceMatrix25_-333-1InformationMeasureCorr2		0.475		334
	Imc1	glcm_Imc11		F4-GrayLevelCooccurenceMatrix25_-333-1InformationMeasureCorr1		0.88		334
	InverseDifference	glcm_Id1		F4-GrayLevelCooccurenceMatrix25_-333-1Homogeneity		0.327		334
		glcm_JointAverage1						
		glcm_Idn1						
		glcm_MCC1						
		glcm_DifferenceAverage1						
		glcm_JointEntropy1						
			YD8GlcmAvDir1Area	F4-GrayLevelCooccurenceMatrix25_-333-1Dissimilarity				
			YD8GlcmAvDir1Entropy	F4-GrayLevelCooccurenceMatrix25_-333-1InverseDiffNorm				
				F4-GrayLevelCooccurenceMatrix25_-333-1SumVariance				
	Contrast	glcm_Contrast4	**YD8GlcmAvDir4Contrast**	F4-GrayLevelCooccurenceMatrix25_-333-4Contrast	**0.922**	0.883	304	331
	Correlation	glcm_Correlation4	YD8GlcmAvDir4Correlat	F4-GrayLevelCooccurenceMatrix25_-333-4Correlation	0.73	0.483	304	331
	SumAverage	glcm_SumAverage4	YD8GlcmAvDir4SumAverg	F4-GrayLevelCooccurenceMatrix25_-333-4SumAverage	0.369	0.341	304	331
	SumSquares	glcm_SumSquares4	YD8GlcmAvDir4SumOfSqs	F4-GrayLevelCooccurenceMatrix25_-333-4Variance	0.848	0.87	304	331
	SumEntropy	glcm_SumEntropy4	YD8GlcmAvDir4SumEntrp	F4-GrayLevelCooccurenceMatrix25_-333-4SumEntropy	0.621	0.773	304	331
	DifferenceEntropy	glcm_DifferenceEntropy4	YD8GlcmAvDir4DifEntrp	F4-GrayLevelCooccurenceMatrix25_-333-4DifferenceEntropy	0.655	0.681	304	331
	DifferenceVariance	glcm_DifferenceVariance4	**YD8GlcmAvDir4DifVarnc**		**0.924**		304	
	InverseDifferenceMoment	glcm_Idm4	YD8GlcmAvDir4InvDfMom	F4-GrayLevelCooccurenceMatrix25_-333-4Homogeneity2	0.841	0.427	304	331
	JointEnergy	glcm_JointEnergy4	YD8GlcmAvDir4AngScMom	F4-GrayLevelCooccurenceMatrix25_-333-4Energy	0.375	0.183	304	331
	ClusterTendency	glcm_ClusterTendency4	**YD8GlcmAvDir4SumVarnc**	**F4-GrayLevelCooccurenceMatrix25_-333-4ClusterTendendcy**	**0.905**	**0.915**	304	331
Grey-level co-occurrence (GLCM) Neighbourhood = 4	ClusterShade	glcm_ClusterShade4		F4-GrayLevelCooccurenceMatrix25_-333-4ClusterShade		0.889		331
	MaxProbability	glcm_MaximumProbability4		F4-GrayLevelCooccurenceMatrix25_-333-4MaxProbability		0.062		331
	Idmn	glcm_Idmn4		F4-GrayLevelCooccurenceMatrix25_-333-4InverseDiffMomentNorm		0.523		331
	InverseVariance	glcm_InverseVariance4		F4-GrayLevelCooccurenceMatrix25_-333-4InverseVariance		0.833		331
	AutoCorrelation	glcm_Autocorrelation4		F4-GrayLevelCooccurenceMatrix25_-333-4AutoCorrelation		0.35		331
	ClusterProminence	glcm_ClusterProminence4		F4-GrayLevelCooccurenceMatrix25_-333-4ClusterProminence		0.889		331
	Imc2	glcm_Imc24		F4-GrayLevelCooccurenceMatrix25_-333-4InformationMeasureCorr2		-0.02		331
	Imc1	glcm_Imc14		F4-GrayLevelCooccurenceMatrix25_-333-4InformationMeasureCorr1		0.718		331
	InverseDifference	glcm_Id4		F4-GrayLevelCooccurenceMatrix25_-333-4Homogeneity		0.541		331
		glcm_JointAverage4						
		glcm_Idn4						
		glcm_MCC4						
		glcm_DifferenceAverage4						
		glcm_JointEntropy4						
			YD8GlcmAvDir4Area	F4-GrayLevelCooccurenceMatrix25_-333-4Dissimilarity				
			YD8GlcmAvDir4Entropy	F4-GrayLevelCooccurenceMatrix25_-333-4InverseDiffNorm				
				F4-GrayLevelCooccurenceMatrix25_-333-4SumVariance				

Supplementary Table 3: Features shared between PyRadiomics, MaZda and IBEX and their linear correlation on a set of HNC ADC maps. Features highlighted with (bold text) have a linear correlation coefficient, $r > 0.901$, (bold text with an asterisk) a linear correlation, $r > 0.965$ and (grey text) unmatched features.

Feature Class	Feature Name				Pearsons Coefficient		Number of Images	
	Shared	*PyRadiomics*	*MaZda*	*IBEX*	*MaZda*	*IBEX*	*MaZda*	*IBEX*
Grey-level co-occurrence (GLCM) Neighbourhood = 7	Contrast	glcm_Contrast7	**YD8GlcmAvDir7Contrast**	F4-GrayLevelCooccurenceMatrix25_-333-7Contrast	**0.908**	0.878	206	320
	Correlation	glcm_Correlation7	YD8GlcmAvDir7Correlat	F4-GrayLevelCooccurenceMatrix25_-333-7Correlation	0.511	0.333	206	320
	SumAverage	glcm_SumAverage7	YD8GlcmAvDir7SumAverg	F4-GrayLevelCooccurenceMatrix25_-333-7SumAverage	0.495	0.381	206	320
	SumSquares	glcm_SumSquares7	YD8GlcmAvDir7SumOfSqs	F4-GrayLevelCooccurenceMatrix25_-333-7Variance	0.763	0.827	206	320
	SumEntropy	glcm_SumEntropy7	YD8GlcmAvDir7SumEntrp	F4-GrayLevelCooccurenceMatrix25_-333-7SumEntropy	0.666	0.767	206	320
	DifferenceEntropy	glcm_DifferenceEntropy7	YD8GlcmAvDir7DifEntrp	F4-GrayLevelCooccurenceMatrix25_-333-7DifferenceEntropy	0.57	0.663	206	320
	DifferenceVariance	glcm_DifferenceVariance7	YD8GlcmAvDir7DifVarnc		0.879		206	
	InverseDifferenceMoment	glcm_Idm7	YD8GlcmAvDir7InvDfMom	F4-GrayLevelCooccurenceMatrix25_-333-7Homogeneity2	0.834	0.49	206	320
	JointEnergy	glcm_JointEnergy7	YD8GlcmAvDir7AngScMom	F4-GrayLevelCooccurenceMatrix25_-333-7Energy	0.316	0.609	206	320
	ClusterTendency	glcm_ClusterTendency7	**YD8GlcmAvDir7SumVarnc**	F4-GrayLevelCooccurenceMatrix25_-333-7ClusterTendendcy	**0.914**	0.877	206	320
	ClusterShade	glcm_ClusterShade7		F4-GrayLevelCooccurenceMatrix25_-333-7ClusterShade		0.832		320
	MaxProbability	glcm_MaximumProbability7		F4-GrayLevelCooccurenceMatrix25_-333-7MaxProbability		0.382		320
	Idmn	glcm_Idmn7		F4-GrayLevelCooccurenceMatrix25_-333-7InverseDiffMomentNorm		0.546		320
	InverseVariance	glcm_InverseVariance7		F4-GrayLevelCooccurenceMatrix25_-333-7InverseVariance		0.683		320
	AutoCorrelation	glcm_Autocorrelation7		F4-GrayLevelCooccurenceMatrix25_-333-7AutoCorrelation		0.38		320
	ClusterProminence	glcm_ClusterProminence7		F4-GrayLevelCooccurenceMatrix25_-333-7ClusterProminence		0.874		320
	Imc2	glcm_Imc27		F4-GrayLevelCooccurenceMatrix25_-333-7InformationMeasureCorr2		-0.18 `		320
	Imc1	glcm_Imc17		F4-GrayLevelCooccurenceMatrix25_-333-7InformationMeasureCorr1		0.673		320
	InverseDifference	glcm_Id7		F4-GrayLevelCooccurenceMatrix25_-333-7Homogeneity		0.6		320
		glcm_JointAverage7						
		glcm_Idn7						
		glcm_MCC7						
		glcm_DifferenceAverage7						
		glcm_JointEntropy7						
			YD8GlcmAvDir7Area	F4-GrayLevelCooccurenceMatrix25_-333-7Dissimilarity				
			YD8GlcmAvDir7Entropy	F4-GrayLevelCooccurenceMatrix25_-333-7InverseDiffNorm				
				F4-GrayLevelCooccurenceMatrix25_-333-7SumVariance				
Grey-level run length (GLRLM)	GrayLevelNonUniformity	glrlm_GrayLevelNonUniformity	**YD8GrlmAvDirGLevNonUn**	F3-GrayLevelRunLengthMatrix25_-333GrayLevelNonuniformity	**0.942**	-0.05	334	334
	RunLengthNonUniformity	glrlm_RunLengthNonUniformity	YD8GrlmAvDirRLNonUni	F3-GrayLevelRunLengthMatrix25_-333RunLengthNonuniformity	0.848	-0.04	334	334
	GrayLevelNonUniformityNormalized	glrlm_GrayLevelNonUniformityNormalizec	YD8GrlmAvDirMGLevNonUn		0.657		334	
	RunLengthNonUniformityNormalize	glrlm_RunLengthNonUniformityNormalize	YD8GrlmAvDirMRLNonUni		0.892		334	
	LongRunEmphasis	glrlm_LongRunEmphasis	**YD8GrlmAvDirLngREmph**	F3-GrayLevelRunLengthMatrix25_-333LongRunEmphasis	**0.906**	-0.19	334	334
	ShortRunEmphasis	glrlm_ShortRunEmphasis	YD8GrlmAvDirShrtREmp	F3-GrayLevelRunLengthMatrix25_-333ShortRunEmphasis	0.893	-0.05	334	334
	RunPercentage	glrlm_RunPercentage	**YD8GrlmAvDirFraction**	F3-GrayLevelRunLengthMatrix25_-333RunPercentage	**0.926**	-0.13	334	334
	ShortRunLowGrayLvlEmp	glrlm_ShortRunLowGrayLevelEmphasis		F3-GrayLevelRunLengthMatrix25_-333ShortRunLowGrayLevelEmpha		0.175		334
	LowGrayLevelRunEmp	glrlm_LowGrayLevelRunEmphasis		F3-GrayLevelRunLengthMatrix25_-333LowGrayLevelRunEmpha		0.213		334
	ShortRunHighGrayLvlEmp	glrlm_ShortRunHighGrayLevelEmphasis		F3-GrayLevelRunLengthMatrix25_-333ShortRunHighGrayLevelEmpha		0.371		334
	LongRunHighGrayLvlEmp	glrlm_LongRunHighGrayLevelEmphasis		F3-GrayLevelRunLengthMatrix25_-333LongRunHighGrayLevelEmpha		0.22		334
	LongRunLowGrayLvlEmp	glrlm_LongRunLowGrayLevelEmphasis		F3-GrayLevelRunLengthMatrix25_-333LongRunLowGrayLevelEmpha		0.395		334
	HighGrayLvlRunEmp	glrlm_HighGrayLevelRunEmphasis		F3-GrayLevelRunLengthMatrix25_-333HighGrayLevelRunEmpha		0.366		334
		glrlm_GrayLevelVariance						
		glrlm_RunVariance						
		glrlm_RunEntropy						
Neighbourhood grey tone difference (NGTDM)	Coarseness	ngtdm_Coarseness		F5-NeighborIntensityDifference25_Coarseness		-0.1		332
	Complexity	ngtdm_Complexity		**F5-NeighborIntensityDifference25_Complexity**		**0.931**		332
	Strength	ngtdm_Strength		F5-NeighborIntensityDifference25_TextureStrength		0.861		332
	Contrast	ngtdm_Contrast		F5-NeighborIntensityDifference25_Contrast		0.891		332
	Busyness	ngtdm_Busyness		F5-NeighborIntensityDifference25_Busyness		-0.05		332

Supplementary Table 4: Features shared between PyRadiomics, MaZda and IBEX and their linear correlation on a set of HNC ADC maps. Features highlighted with (bold text) have a linear correlation coefficient, $r > 0.901$, (bold text with an asterisk) a linear correlation, $r > 0.965$ and (grey text) unmatched features

Supplementary Table 5: Intensity histogram feature equation comparison. Author comments specific to each feature are annotated by italic font.

	**PyRadiomics**	**IBEX**	**MaZda**		
General Description of Feature class & Shared equation terms	First-order statistics describe the distribution of voxel intensities within the image region defined by the mask through commonly used and basic metrics.<br>Let:<br>• $\mathbf{X}$ be a set of N_p voxels included in the ROI<br>• $\mathbf{P}(i)$ be the first order histogram with N_g discrete intensity levels, where N_g is the number of non-zero bins, equally spaced from 0 with a width defined in the binWidth<br>$p(i)$ be the normalized first order histogram and equal to $\frac{\mathbf{P}(i)}{N_p}$	-Description:<br>1. This method is to compute histogram from image inside the binary mask.<br>2. Histogram is passed into IntensityHistogram_Feature.m to compute the related features.<br><br>-Parameters:<br>1. NBins: The number of bins.<br>2. RangeMin: Lower bound of bin location.<br>3. RangeMax: Upper bound of bin location.<br>4. RangeFix: 1==The specified RangeMin and RangeMax specified are used.<br>0==Ignore the specified RangeMin and RangeMax, and<br>RangeMin and RangeMax are dynamically determined by min and max of the current image.<br>5. OnlyUseMaxSlice: 1: Binary mask only contains the binary slice with the maximum area. 0: Use the binary mask as it is.	The $p(k)$ is the normalized histogram function.<br>$p(k) = \frac{1}{Area} \sum_{(x,y) \in ROI} \begin{cases} 1 : I(x,y) = k \\ 0 : I(x,y) \neq k \end{cases}$<br>Parameter $\Theta = 2^n - 1$ represents maximum grey level, n is a number of bits per pixel, $I(.)$ is an image, and (x, y) are pixel coordinates.<br>$Area = \sum_{(x,y) \in ROI} 1$		
Source	PyRadiomics (2.1.0) Documentation https://pyradiomics.readthedocs.io/en/2.1.0/features.html#	IBEX software help dialogues (pop-up text descriptions available per feature, often citing an article(s) for feature details)	Qmazda Documentation http://www.eletel.p.lodz.pl/pms/Programy/qmazda.pdf		
**Shared Feature Name**	**PyRadiomics**	**IBEX**	**MaZda**	**IBEX Correl.**	**MaZda Correl.**
Skewness	$skewness = \frac{\mu_3}{\sigma^3} = \frac{\frac{1}{N_p}\sum_{i=1}^{N_p}(\mathbf{X}(i)-\bar{X})^3}{\left(\sqrt{\frac{1}{N_p}\sum_{i=1}^{N_p}(\mathbf{X}(i)-\bar{X})^2}\right)^3}$	Measure the asymmetry of the occurence probability values in the histogram.<br><br>*No equation details*	$Skewness = Variance^{-\frac{3}{2}} \left(\sum_{k=0}^{\Theta} (k-\mu)^3 p(k) \right)$<br>$Variance = \sum_{k=0}^{\Theta} (k-\mu)^2 p(k)$	0.892	-0.987

			Looks different… iterating over grey levels rather than voxels in ROI. May be equivalent? Seems to have a reversed/negative skew		
10Percentile	The 10th percentile of $\mathbf{X}$	Percentiles of the occurence probability values in the histogram.	$Perc10 = min(K): \sum_{k=0}^{K} p(k) \geq 0{,}10$	0.974	0.967
90Percentile	The 90th percentile of $\mathbf{X}$	Percentiles of the occurence probability values in the histogram.	$Perc90 = min(K): \sum_{k=0}^{K} p(k) \geq 0{,}90$	0.994	0.976
Kurtosis	$kurtosis = \frac{\mu_4}{\sigma^4} = \frac{\frac{1}{N_p}\sum_{i=1}^{N_p}(\mathbf{X}(i)-\bar{X})^4}{\left(\frac{1}{N_p}\sum_{i=1}^{N_p}(\mathbf{X}(i)-\bar{X})^2\right)^2}$	Measure the peakedness of the occurence probability values in the histogram.<br><br>*No equation details*	$Kurtosis = Variance^{-2}\left(\sum_{k=0}^{\Theta}(k-\mu)^4 p(k)\right) - 3$<br>$Variance = \sum_{k=0}^{\Theta}(k-\mu)^2 p(k)$<br>*Looks different… iterating over grey levels rather than voxels in ROI … impact of -3?*	0.832	0.977
Variance	$variance = \frac{1}{N_p}\sum_{i=1}^{N_p}(\mathbf{X}(i)-\bar{X})^2$		$Variance = \sum_{k=0}^{\Theta}(k-\mu)^2 p(k)$<br>*Looks different… iterating over grey levels rather than voxels in ROI. May be equivalent?*		0.999
Mean	$mean = \frac{1}{N_p}\sum_{i=1}^{N_p}\mathbf{X}(i)$		$Mean = \mu = \sum_{k=0}^{\Theta} k\, p(k)$<br>*Different way of calculating the same thing*		1
Interquartile Range	$interquartile\ range = \mathbf{P}_{75} - \mathbf{P}_{25}$	The interquartile range of the occurence probability values in the histogram.		0.963	

MeanAbsDev	$MAD = \frac{1}{N_p}\sum_{i=1}^{N_p}\lvert\mathbf{X}(i) - \bar{X}\rvert$	The mean absolute deviation of the occurence probability values in the histogram.		0.97	
Range	$range = \max(\mathbf{X}) - \min(\mathbf{X})$	Measure the range(MaxValue-MinValue) of the occurence probability values in the histogram.		0.91	

Supplementary Table 6: Shape feature equation comparison. Author comments specific to each feature are annotated by italic font.

	**PyRadiomics**	**IBEX**	**MaZda**		
General Description of Feature class & Shared equation terms	Unless otherwise specified, features are derived from the approximated shape defined by the triangle mesh. To build this mesh, vertices (points) are first defined as points halfway on an edge between a voxel included in the ROI and one outside the ROI. By connecting these vertices a mesh of connected triangles is obtained, with each triangle defined by 3 adjacent vertices, which shares each side with exactly one other triangle.<br>This mesh is generated using a marching cubes algorithm. In this algorithm, a 2x2 cube is moved through the mask space. For each position, the corners of the cube are then marked 'segmented' (1) or 'not segmented' (0). Treating the corners as specific bits in a binary number, a unique cube-index is obtained (0-255). This index is then used to determine which triangles are present in the cube, which are defined in a lookup table.<br>These triangles are defined in such a way, that the normal (obtained from the cross product of vectors describing 2 out of 3 edges) are always oriented in the same direction. For PyRadiomics, the calculated normals are always pointing outward. This is necessary to obtain the correct signed volume used in calculation of `MeshVolume`.<br>Let:<br>• N_v represent the number of voxels included in the ROI<br>• N_f represent the number of faces (triangles) defining the Mesh.<br>• V the volume of the mesh in mm^3, calculated by `getMeshVolumeFeatureValue()`<br>• A the surface area of the mesh in mm^2, calculated by `getMeshSurfaceAreaFeatureValue()`<br>References:<br>• Lorensen WE, Cline HE. Marching cubes: A high resolution 3D surface construction algorithm. ACM SIGGRAPH Comput Graph Internet. 1987;21:163-9.<br><br>An ROI enclosing ellipsoid is performed with principle component analysis (PCA)<br>This feature yield the largest axis length of the ROI-enclosing ellipsoid and is calculated using the largest principal component λ_{major}.<br>The principal component analysis is performed using the physical coordinates of the voxel centers defining the ROI. It therefore takes spacing into account, but does not make use of the shape mesh.<br>This feature yield the second-largest axis length of the ROI-enclosing ellipsoid and is calculated using the largest principal component λ_{minor}.	-Description:<br>1. This method is just a layer on Shape_Feature.m.<br>2. Image and binary mask are passed into Shape_Feature.m to compute the related features.<br><br>-Parameters:<br>No<br><br>-Revision:<br>2014-01-01: The method is implemented.<br>2014-07-28: Feature SurfaceArea, SurfaceAreaDensity, MeanBreath, and VoxelSize are added.	In MaZda papers (2009, 2017) reference a book for morphological analysis [unable to locate a copy]<br>Luerkens, D. W. (1991). *Theory and application of morphological analysis: fine particles and surfaces* (Vol. 5). CRC press.<br><br>The features reported have the prefix MorItk (assuming morphological and ITK as the calculating software), looking through the ITK documentation the most likely C++ class was LabelObject:<br>https://itk.org/Doxygen/html/classitk_1_1LabelObject.html<br>Which references<br>Lehmann, G. (2007). Label object representation and manipulation with ITK. *Insight J*, *8*, 1-31.<br><br>*This is not a very strong link*		
Source	PyRadiomics (2.1.0) Documentation https://pyradiomics.readthedocs.io/en/2.1.0/features.html#	IBEX software help dialogues (pop-up text descriptions available per feature, often citing an article(s) for feature details)<br>Aerts, Hugo JWL, et al. "Decoding tumour phenotype by noninvasive imaging using a quantitative radiomics approach." Nature communications 5.1 (2014): 1-9.<br>Legland, David, Kiên Kiêu, and Marie-Françoise Devaux. "Computation of Minkowski measures on 2D and 3D binary images." Image Analysis & Stereology 26.2 (2007): 83-92.	Qmazda Documentation<br>http://www.eletel.p.lodz.pl/pms/Programy/qmazda.pdf<br>*(no specific feature detail)*<br><br>Lehmann, G. (2007). Label object representation and manipulation with ITK. *Insight J*, *8*, 1-31.		

Shared Feature Name	PyRadiomics	IBEX	MaZda	IBEX Correl.	MaZda Correl.
VoxelVolume	$V_{voxel} = \sum_{k=1}^{N_v} V_k$<br>*Sum the volume of each voxel in the ROI to get the volume of the ROI*	"VoxelSize": The physical voxel size.<br><br>*Name similarity is ambiguous as could refer to the size of a voxel, or the size of the volume based on voxels. Not included in final analysis due to equation/description and poor correlation.*	*Size was selected for name similarity to Volume. "SizeX" feature was selected as there is no general Size feature, and it had the highest correlation of (SizeX, SizeY, SizeZ). Moderate correlation would suggest size relates to the size of the voxel ROI, rather than the size of an individual voxel dimension.*		0.836
Max3D Diameter / FeretDiameter	Maximum 3D diameter is defined as the largest pairwise Euclidean distance between tumor surface mesh vertices.<br>Also known as Feret Diameter.	Max3DDiameter= largest pairwise Euclidean distance between voxels on the surface of the tumor volume.	*FeretDiameter* is the diameter in physical units of the sphere which include all the object. The feret diameter is not computed by default, because of its high computation. Its type is *double*.	0.996	0.858
MeshVolume	$V_i = \frac{Oa_i \cdot (Ob_i \times Oc_i)}{6}$ (1)<br>$V = \sum_{i=1}^{N_f} V_i$ (2)<br>The volume of the ROI V is calculated from the triangle mesh of the ROI. For each face i in the mesh, defined by points a_i, b_i and c_i, the (signed) volume V_f of the tetrahedron defined by that face and the origin of the image (O) is calculated. (1) The sign of the volume is determined by the sign of the normal, which must be consistently defined as either facing outward or inward of the ROI.<br>Then taking the sum of all V_i, the total volume of the ROI is obtained (2)	Volume:<br>The physical volume treating the edge voxels differently.<br>-Parameters:<br>1. EdgeVoxelFraction: edge voxel is considered as EdgeVoxelFraction*Voxel. [Defaults to 0.5]<br><br>*Excellent correlation, even though using a non-mesh based metric*	a is the measured area of the object[5].<br>[5]More details about perimeter estimation wil<br>*["Area" was moderately correlated with Mesh Volume]*	0.999	0.848
Sphericity	$sphericity = \frac{\sqrt[3]{36\pi V^2}}{A}$<br>This feature is correlated to Compactness 1, Compactness 2 and Spherical Disproportion.	$sphericity = \frac{\pi^{\frac{1}{3}}(6V)^{\frac{2}{3}}}{A}$<br>From: Aerts 2014	*"Roundness" sounded similar to sphericity and had a good correlation.*<br>$R = \frac{A_n(r)}{a}$ (15)<br>where R is the roundness, a is the measured area of the object[5], and the r is the radius of an hypersphere with the same volume than the object, computed using equation 11.<br>$A_n(r) = \frac{nV_n}{r}$<br>a is the measured area of the object[5].<br>[5]More details about perimeter estimation will be published in another article.<br>$V_n(r) = \frac{\pi^{\frac{n}{2}} r^n}{\Gamma(\frac{n}{2}+1)}$<br>where V_n is the volume of the hypersphere n is the image dimens<br>$\Gamma\left(\frac{n}{2}+1\right) = \begin{cases} (\frac{n}{2})! & \text{if } n \text{ is} \\ \sqrt{\pi}\frac{n!!}{2^{(n+1)/2}} & \text{if } n \text{ is} \end{cases}$<br>*Hard to establish equation similarity*	0.899	0.925

Elongation	Elongation shows the relationship between the two largest principal components in the ROI shape. For computational reasons, this feature is defined as the inverse of true elongation.<br>$elongation = \sqrt{\frac{\lambda_{minor}}{\lambda_{major}}}$<br>Here, λ_{major} and λ_{minor} are the lengths of the largest and second largest principal component axes. The values range between 1 (where the cross section through the first and second largest principal moments is circle-like (non-elongated)) and 0 (where the object is a maximally elongated: i.e. a 1 dimensional line).	$spherical\ disproportion = \frac{A}{4\pi R^2}$<br>Where R is the radius of a sphere with the same volume as the tumor.<br>From: Aerts 2014<br><br>*Different equation, though still a measure of disproportion between a sphere and an ellipse (just an areas based one)*	*BinaryElongation* is the elongation of the shape, computed as the ratio of the largest principal moment by the smallest principal moment. Its value is greater or equal to 1. Its type is *double*.<br>*Sounds like a similar approach, but may be the inverse (and no mention of a sqrt)*		-0.021
SurfaceArea	$A_i = \frac{1}{2}\lvert \text{a}_i\text{b}_i \times \text{a}_i\text{c}_i \rvert$ (1)<br>$A = \sum_{i=1}^{N_f} A_i$ (2)<br>a_ib_i and a_ic_i are edges of the ith triangle in the mesh, formed by vertices a_i, b_i and c_i.<br>*Sum the area of each triangular face*	$S_{\text{d}}(x) = 4\sum_k \frac{c_k}{\lambda(L_k)} \sum_{C \in \mathscr{X}_{\text{r},k}(x)} (-1)^{\dim C} \frac{\alpha(C,x)}{4\pi}$, (14)<br>where $\lambda(L_k)$ is the distance between two pixels on a discrete line oriented in the k-th direction. The final<br>where $\alpha(X_{\text{r}}, x)$ is the normal angle of X_{r} at x, defined as the solid angle of the set of vectors opposite to the tangent cone, and s_d is the surface of the unit ball in dimension d.<br>From: Legland 2007<br><br>*Difficult to assess equation similarity, correlation is very high*	• *EquivalentPerimeter* is the equivalent perimeter of the hypersphere of the same size than the label object. The value depends on the image spacing. Its type is *double*.<br>• *EquivalentEllipsoidPerimeter* is the size of the ellipsoid of the same size and the same ratio on all the axes than the label object. The value depends on the image spacing. Its type is *itk::Vector¡ double, ImageDimension ¿*.<br>*A perimeter in 3D can be considered a surface area, there is no mention of a mesh based approach in MaZDa though.*	0.998	0.922
Surface VolumeRatio	$surface\ to\ volume\ ratio = \frac{A}{V}$<br>*Based on the mesh surface area and mesh volume*	SurfaceAreaDensity == SurfaceVolumeRation in Hugo's paper below.<br>-Formula: SurfaceAreaDensity= (surface area of the binary mask)/(volume of the binary mask).<br>$surface\ to\ volume\ ratio = \frac{A}{V}$<br>The volume ($V$) of the tumor is<br>the tumor region and multiplying		0.985	

		$$A = \sum_{i=1}^{N} \frac{1}{2} \lvert \mathbf{a}_i \mathbf{b}_i \times \mathbf{a}_i \mathbf{c}_i \rvert$$<br>From: Aerts 2014<br><br>*Name similarity, also has equation similarity, though using volume based on voxels (not a mesh)*			
Flatness	Flatness shows the relationship between the largest and smallest principal components in the ROI shape. For computational reasons, this feature is defined as the inverse of true flatness.<br><br>$$flatness = \sqrt{\frac{\lambda_{least}}{\lambda_{major}}}$$<br><br>Here, λ_{major} and λ_{least} are the lengths of the largest and smallest principal component axes. The values range between 1 (non-flat, sphere-like) and 0 (a flat object, or single-slice segmentation).	"Roundness"<br>1. Measure how much the binary mask is close to circle in 2D.<br>2. Refer to MATLAB "regionprops(Mask, 'Eccentricity')" for details<br>-Formula:<br>1. First, compute roundness value in 2D slice-by-slice. Roundness=1-regionprops(2DMask, 'Eccentricity')<br>2. Then, compute the mean of roundness value among the slices.<br><br>`'Eccentricity'` Eccentricity of the ellipse that has the same second-moments as the region, returned as a scalar. The eccentricity is the ratio of the distance between the foci of the ellipse and its major axis length. The value is between 0 and 1. (0 and 1 are degenerate cases. An ellipse whose eccentricity is 0 is actually a circle, while an ellipse whose eccentricity is 1 is a line segment.)<br>https://www.mathworks.com/help/images/ref/regionprops.html<br>*Hard to assess equation similarity. The values of both are meant to reflect a spectrum of flatness to sphere-like/roundness.*		0.462	

Supplementary Table 7: GLCM feature equation comparison. Author comments specific to each feature are annotated by italic font. Correlations are for GLCM features calculated with a neighbourhood of 1 as this has the most comparable directions/angles between the three software.

	**PyRadiomics**	**IBEX**	**MaZda**		
General Description of Feature class & Shared equation terms	ϵ be an arbitrarily small positive number ($\approx 2.2 \times 10^{-16}$)<br>$\mathbf{P}(i,j)$ be the co-occurence matrix for an arbitrary δ and θ<br>$p(i,j)$ be the normalized co-occurence matrix and equal to $\frac{\mathbf{P}(i,j)}{\sum \mathbf{P}(i,j)}$<br>$N_g$ be the number of discrete intensity levels in the image<br>$p_x(i) = \sum_{j=1}^{N_g} P(i,j)$ be the marginal row probabilities<br>$p_y(j) = \sum_{i=1}^{N_g} P(i,j)$ be the marginal column probabilities<br>μ_x be the mean gray level intensity of p_x and defined as $\mu_x = \sum_{i=1}^{N_g} p_x(i)i$<br>$\mu_y$ be the mean gray level intensity of p_y and defined as $\mu_y = \sum_{j=1}^{N_g} p_y(j)j$<br>$\sigma_x$ be the standard deviation of p_x<br>σ_y be the standard deviation of p_y<br>$p_{x+y}(k) = \sum_{i=1}^{N_g} \sum_{j=1}^{N_g} p(i,j),\ \text{where } i+j=k, \text{ and } k=2,3,\dots,2N_g$<br>$p_{x-y}(k) = \sum_{i=1}^{N_g} \sum_{j=1}^{N_g} p(i,j),\ \text{where } \lvert i-j \rvert=k, \text{ and } k=0,1,\dots,N_g-1$<br>$HX = -\sum_{i=1}^{N_g} p_x(i) \log_2 \big(p_x(i)+\epsilon\big)$ be the entropy of p_x<br>$HY = -\sum_{j=1}^{N_g} p_y(j) \log_2 \big(p_y(j)+\epsilon\big)$ be the entropy of p_y<br>$HXY = -\sum_{i=1}^{N_g} \sum_{j=1}^{N_g} p(i,j) \log_2 \big(p(i,j)+\epsilon\big)$ be the entropy of $p(i,j)$<br>$HXY1 = -\sum_{i=1}^{N_g} \sum_{j=1}^{N_g} p(i,j) \log_2 \big(p_x(i)p_y(j)+\epsilon\big)$<br>$HXY2 = -\sum_{i=1}^{N_g} \sum_{j=1}^{N_g} p_x(i)p_y(j) \log_2 \big(p_x(i)p_y(j)+\epsilon\big)$	Haralick 1973 (Soh 1999 refs these also)<br>$p(i,j)$ (i,j)th entry in a normalized gray-tone spatial-dependence matrix, $= P(i,j)/R$.<br>$p_x(i)$ ith entry in the marginal-probability matrix obtained by summing the rows of $p(i,j)$, $= \sum_{j=1}^{N_g} P(i,j)$.<br>$N_g$ Number of distinct gray levels in the quantized image.<br>$\sum_i$ and $\sum_j$ $\sum_{i=1}^{N_g}$ and $\sum_{j=1}^{N_g}$, respectively.<br>$p_y(j) = \sum_{i=1}^{N_g} p(i,j).$<br>$p_{x+y}(k) = \sum_{\substack{i=1 \\ i+j=k}}^{N_g} \sum_{j=1}^{N_g} p(i,j), \quad k = 2,3,\cdots,2N_g.$<br>$p_{x-y}(k) = \sum_{\substack{i=1 \\ \lvert i-j \rvert=k}}^{N_g} \sum_{j=1}^{N_g} p(i,j), \quad k = 0,1,\cdots,N_g-1.$<br>Aerts 2014 (supp info)<br>A GLCM is defined as $\mathbf{P}(i,j;\delta,\alpha)$, a matrix with size $N_g \times N_g$ describing the second-order joint probability function of an image, where the (i,j)th element represents the number of times the combination of intensity levels i and j occur in two pixels in the image, that are separated by a distance of δ pixels in direction α, and N_g is the number of discrete gray level intensities. As a two dimensional example, let the following matrix<br>$\mathbf{P}(i,j)$ be the co-occurrence matrix for an arbitrary δ and α,<br>N_g be the number of discrete intensity levels in the image,<br>μ be the mean of $\mathbf{P}(i,j)$,<br>$p_x(i) = \sum_{j=1}^{N_g} \mathbf{P}(i,j)$ be the marginal row probabilities,<br>$p_y(i) = \sum_{i=1}^{N_g} \mathbf{P}(i,j)$ be the marginal column probabilities,<br>μ_x be the mean of p_x,<br>μ_y be the mean of p_y,<br>σ_x be the standard deviation of p_x,<br>σ_y be the standard deviation of p_y,<br>$p_{x+y}(k) = \sum_{i=1}^{N_g} \sum_{j=1}^{N_g} P(i,j),\ i+j=k,\ k=2,3,\dots,2N_g,$<br>$p_{x-y}(k) = \sum_{i=1}^{N_g} \sum_{j=1}^{N_g} P(i,j),\ \lvert i-j \rvert=k,\ k=0,1,\dots,N_g-1,$<br>$HX = -\sum_{i=1}^{N_g} p_x(i) \log_2[p_x(i)]$ be the entropy of p_x,<br>$HY = -\sum_{i=1}^{N_g} p_y(i) \log_2[p_y(i)]$ be the entropy of p_y,<br>$H = -\sum_{i=1}^{N_g} \sum_{j=1}^{N_g} P(i,j) \log_2[P(i,j)]$ be the entropy of $P(i,j)$,<br>$HXY1 = -\sum_{i=1}^{N_g} \sum_{j=1}^{N_g} \mathbf{P}(i,j) \log(p_x(i)p_y(j)),$<br>$HXY2 = -\sum_{i=1}^{N_g} \sum_{j=1}^{N_g} p_x(i)p_y(j) \log(p_x(i)p_y(j)).$	The co-occurrence matrix holds counts of co-occurrences of pixels having some specified gray-levels. The pairs of pixels are considered, such that one of the pixels is situated at the offset (Δx, Δy) from the other one. The co-occurrence matrix (asymmetric) is defined as:<br>$C_{\Delta x,\Delta y}(i,j) = \sum_{(x,y)\in ROI} \begin{cases} 1 : I(x,y)=i \wedge I(x+\Delta x, y+\Delta x)=j \\ 0 : otherwise \end{cases}$<br>Optionally the co-occurrence matrix (symmetric) is defined as:<br>$C_{\Delta x,\Delta y}(i,j) = \sum_{(x,y)\in ROI} \begin{cases} 1 : (I(x,y)=i \wedge I(x+\Delta x, y+\Delta x)=j) \vee (I(x+\Delta x, y+\Delta x)=i \wedge I(x,y)=j) \\ 0 : otherwise \end{cases}$<br>The size of the matrix in both the cases is $2^n \times 2^n$ where n is a number of bits per pixel.<br>The normalized matrix, or probability of co-occurrence,<br>$p_{\Delta x,\Delta y}(k,l) = \frac{C_{\Delta x,\Delta y}(k,l)}{A}$<br>where $A = \sum_{k=0}^{\Theta} \sum_{l=0}^{\Theta} C_{\Delta x,\Delta y}(k,l)$<br>where:<br>$\Theta = 2^n - 1$<br>$\mu_k = \sum_{k=0}^{\Theta} \sum_{l=0}^{\Theta} k\, p(k,l)$<br>$\mu_l = \sum_{k=0}^{\Theta} \sum_{l=0}^{\Theta} l\, p(k,l)$<br>$\sigma_k = \sum_{k=0}^{\Theta} \sum_{l=0}^{\Theta} (k-\mu_k)^2 p(k,l)$<br>$\sigma_l = \sum_{k=0}^{\Theta} \sum_{l=0}^{\Theta} (l-\mu_l)^2 p(k,l)$<br>$p_{sum}(m) = \sum_{k=0}^{m-1} p(k, m-k)$<br>$p_{dif}(m) = \begin{cases} \sum_{k=0}^{\Theta-m} \big(p(k,m+k)+p(m+k,k)\big), & m \neq 0 \\ \sum_{k=0}^{\Theta-m} \big(p(k,k)\big), & m=0 \end{cases}$<br>$\mu_{dif} = \sum_{m=0}^{2\Theta} m\, p_{dif}(m)$<br>*Uses 0 indexing (0,Ng-1), PyRad and IBEX use 1 indexing (1, Ng)*<br>*P_{sum} and P_{diff} have a different notation than PyRad/IBEX but may be identical*		
Source	PyRadiomics (2.1.0) Documentation https://pyradiomics.readthedocs.io/en/2.1.0/features.html#	IBEX software help dialogues	Qmazda Documentation http://www.eletel.p.lodz.pl/pms/Programy/qmazda.pdf		

		(pop-up text descriptions available per feature, often citing an article(s) for feature details)<br><br>Aerts, Hugo JWL, et al. "Decoding tumour phenotype by noninvasive imaging using a quantitative radiomics approach." Nature communications 5.1 (2014): 1-9.<br><br>Haralick, Robert M., Karthikeyan Shanmugam, and Its' Hak Dinstein. "Textural features for image classification." IEEE Transactions on systems, man, and cybernetics 6 (1973): 610-621.<br><br>Soh, L-K., and Costas Tsatsoulis. "Texture analysis of SAR sea ice imagery using gray level co-occurrence matrices." IEEE Transactions on geoscience and remote sensing 37.2 (1999): 780-795.			

**Shared Feature Name**	**PyRadiomics**	**IBEX**	**MaZda**	**IBEX Correl.**	**MaZda Correl.**
Contrast	$contrast = \sum_{i=1}^{N_g}\sum_{j=1}^{N_g}(i-j)^2 p(i,j)$	*2) Contrast:*<br>$f_2 = \sum_{n=0}^{N_g-1} n^2 \left\{ \sum_{i=1}^{N_g} \sum_{j=1}^{N_g} p(i,j) \right\}, \; \lvert i-j \rvert = n.$<br>From: Haralick 1973	$Contrast = \sum_{m=1}^{2^{n-1}} \left(m^2 p_{dif}(m) \right)$<br>Different? Still good correlation	0.924	0.963
Correlation	$correlation = \frac{\sum_{i=1}^{N_g}\sum_{j=1}^{N_g} p(i,j)ij - \mu_x\mu_y}{\sigma_x(i)\sigma_y(j)}$	*3) Correlation:*<br>$f_3 = \frac{\sum_i \sum_j (ij)p(i,j) - \mu_x\mu_y}{\sigma_x\sigma_y}$<br>From: Haralick 1973	$Correlat = \frac{1}{\rho_k \rho_l} \sum_{k=0}^{\Theta} \sum_{l=0}^{\Theta} \left((k+1)(l+1)p(k,l) - \mu_k \mu_l \right)$<br>Difference iteration of p(k,l)	0.862	0.777
SumAverage	$sum\ average = \sum_{k=2}^{2N_g} p_{x+y}(k)k$<br>• $p_{x+y}(k) = \sum_{i=1}^{N_g}\sum_{j=1}^{N_g} p(i,j)$, where $i+j=k$, and $k = 2,3,\ldots,2N_g$	*6) Sum Average:*<br>$f_6 = \sum_{i=2}^{2N_g} i p_{x+y}(i).$<br>$p_{x+y}(k) = \sum_{i=1}^{N_g} \sum_{j=1}^{N_g} p(i,j), \; i+j=k, \quad k = 2,3,\cdots,2N_g.$<br>From: Haralick 1973<br>$sum\ average = \sum_{i=2}^{2N_g} [i\mathbf{P}_{x+y}(i)]$	$SumAverg = \sum_{m=1}^{2^{n+1}} \left(m\, p_{sum}(m) \right)$<br>Equations look identical, but correlation is poor	0.361	0.38

		From: Aerts 2014<br>*Equations look identical, but correlation is poor*			
SumSquares	$sum\ squares = \sum_{i=1}^{N_g}\sum_{j=1}^{N_g}(i-\mu_x)^2 p(i,j)$	**4) *Sum of Squares:* Variance**<br>$f_4 = \sum_i \sum_j (i - \mu)^2 p(i,j).$<br>From: Haralick 1973<br>*IBEX documentation references*<br>$variance = \sum_{i=1}^{N_g}\sum_{j=1}^{N_g}(i-\mu)^2 \mathbf{P}(i,j)$<br>From: Aerts 2014	$SumOfSqs = \sum_{k=1}^{N_g}\sum_{l=1}^{N_g}(k-\mu_k)^2 p(k,l)$	0.941	0.901
SumEntropy	$sum\ entropy = \sum_{k=2}^{2N_g} p_{x+y}(k)\log_2(p_{x+y}(k)+\epsilon)$<br>• $p_{x+y}(k) = \sum_{i=1}^{N_g}\sum_{j=1}^{N_g} p(i,j)$, where $i+j=k$, and $k = 2,3,\ldots,2N_g$	***8) Sum Entropy:*** [2]<br>$f_8 = -\sum_{i=2}^{2N_g} p_{x+y}(i)\log\{p_{x+y}(i)\}.$<br>$p_{x+y}(k) = \sum_{\substack{i=1\\i+j=k}}^{N_g}\sum_{j=1}^{N_g} p(i,j), \quad k = 2,3,\cdots,2N_g.$<br>[2] Since some of the probabilities may be zero, and log (0) is not defined, it is recommended that the term log $(p + \epsilon)$ (ϵ an arbitrarily small positive constant) be used in place of log (p) in entropy computations.<br>*Negative sign does not match PyRadiomics*<br>From: Haralick 1973	$SumEntrp = -\sum_{m=1}^{2^{n+1}} p_{sum}(m)\log(p_{sum}(m))$<br>*Negative sign does not match PyRadiomics*<br>*Range(1, 2^{n+1}) is equivalent to (2, $2N_g$)*	0.679	0.709
Difference Entropy	$difference\ entropy = \sum_{k=0}^{N_g-1} p_{x-y}(k)\log_2(p_{x-y}(k)+\epsilon)$<br>$p_{x-y}(k) = \sum_{i=1}^{N_g}\sum_{j=1}^{N_g} p(i,j)$, where $\lvert i-j\rvert = k$, and $k = 0,1,\ldots,N_g-1$	***11) Difference Entropy:***<br>$f_{11} = -\sum_{i=0}^{N_g-1} p_{x-y}(i)\log\{p_{x-y}(i)\}.$<br>$p_{x-y}(k) = \sum_{\substack{i=1\\\lvert i-j\rvert=k}}^{N_g}\sum_{j=1}^{N_g} p(i,j), \quad k = 0,1,\cdots,N_g-1.$<br>[2] Since some of the probabilities may be zero, and log (0) is not defined, it is recommended that the term log $(p + \epsilon)$ (ϵ an arbitrarily small positive constant) be used in place of log (p) in entropy computations.<br>*Negative sign does not match PyRadiomics*<br>From: Haralick 1973	$DifEntrp = -\sum_{m=1}^{2^n} p_{dif}(m)\log(p_{dif}(m))$<br>*Range(1, 2^n) is equivalent to (0, N_g - 1)*<br>*Negative sign does not match PyRadiomics*	0.697	0.849
Difference Variance	$difference\ variance = \sum_{k=0}^{N_g-1}(k-DA)^2 p_{x-y}(k)$<br>$difference\ average = \sum_{k=0}^{N_g-1} k p_{x-y}(k)$<br>*Assuming DA is difference average	***10) Difference Variance:***<br>$f_{10} = \text{variance of } p_{x-y}.$<br>$p_{x-y}(k) = \sum_{\substack{i=1\\\lvert i-j\rvert=k}}^{N_g}\sum_{j=1}^{N_g} p(i,j), \quad k = 0,1,\cdots,N_g-1.$<br>*From: Haralick 1973, not calculated by IBEX*	$DifVarnc = \sum_{m=1}^{\Theta}(i-\mu_{dif})^2 p_{dif}(m)$<br>$\mu_{dif} = \sum_{m=0}^{2\Theta} m\, p_{dif}(m)$<br>*Different range (1, 2^n-1) vs PyRad (0, Ng-1)*<br>*Different calculation range for DA/u_{diff}*<br>*good correlation*		0.956

Inverse Difference Moment	$IDM = \sum_{k=0}^{N_g-1} \frac{p_{x-y}(k)}{1+k^2}$<br>$p_{x-y}(k) = \sum_{i=1}^{N_g}\sum_{j=1}^{N_g} p(i,j), \text{ where } \lvert i-j\rvert = k, \text{ and } k = 0,1,\ldots,N_g-1$	5) ***Inverse Difference Moment:***<br>$f_5 = \sum_i \sum_j \frac{1}{1+(i-j)^2} p(i,j).$<br>From: Haralick 1973<br>$homogeneity\ 2 = \sum_{i=1}^{N_g}\sum_{j=1}^{N_g} \frac{\mathbf{P}(i,j)}{1+\lvert i-j\rvert^2}$<br>From: Aerts 2014<br>*A.K.A. Homogeneity 2 as per PyRadiomics docs.*<br>*Different numerator, poor correlation*	$InvDfMom = \sum_{k=0}^{\Theta}\sum_{l=0}^{\Theta} \frac{p(k,l)}{1+(k-l)^2}$<br>*Different numerator , good correlation*	0.241	0.933
Inverse Difference	$ID = \sum_{k=0}^{N_g-1} \frac{p_{x-y}(k)}{1+k}$	$homogeneity\ 1 = \sum_{i=1}^{N_g}\sum_{j=1}^{N_g} \frac{\mathbf{P}(i,j)}{1+\lvert i-j\rvert}$<br>From:Aerts 2014<br>*(labelled as such in IBEX dialogue)*		0.327	
Max Probability	$maximum\ probability = \max\left(p(i,j)\right)$	$f_{10} = \underset{i,j}{\mathrm{MAX}}\, p(i,j).$<br>From: Soh 1999<br>*Identical equation, but poor correlation*		0.027	
Idmn (Inverse Difference Moment Normalised)	$IDMN = \sum_{k=0}^{N_g-1} \frac{p_{x-y}(k)}{1+\left(\frac{k^2}{N_g^2}\right)}$<br>$p_{x-y}(k) = \sum_{i=1}^{N_g}\sum_{j=1}^{N_g} p(i,j), \text{ where } \lvert i-j\rvert = k, \text{ and } k = 0,1,\ldots,N_g-1$	$IDMN = \sum_{i=1}^{N_g}\sum_{j=1}^{N_g} \frac{\mathbf{P}(i,j)}{1+\left(\frac{\lvert i-j\rvert^2}{N^2}\right)}$<br>From: Aerts 2014<br>*Information dialogue references Haralick 1973 (couldn't find eqn)*<br>*Uses P not P_{x-y}, different iteration (equiv?)*		0.348	
ClusterShade	$cluster\ shade = \sum_{i=1}^{N_g}\sum_{j=1}^{N_g} \left(i+j-\mu_x-\mu_y\right)^3 p(i,j)$	$f_8 = \sum_i \sum_j (i+j-\mu_x-\mu_y)^3 p(i,j).$<br>From: Soh 1999		0.882	
JointEnergy	$joint\ energy = \sum_{i=1}^{N_g}\sum_{j=1}^{N_g} \left(p(i,j)\right)^2$	***Angular Second Moment:***<br>$f_1 = \sum_i \sum_j \{p(i,j)\}^2.$<br>From: Haralick 1973<br>*(no joint energy but this equation matched)*	$AngScMom = \sum_{k=0}^{\Theta}\sum_{l=0}^{\Theta} p^2(k,l)$<br>*Feature missed in name comparison, but identified due to equation similarity*	0.044	0.282

		$$energy = \sum_{i=1}^{N_g}\sum_{j=1}^{N_g}[\mathbf{P}(i,j)]^2$$<br>From: Aerts 2014<br>*(not referenced in IBEX for this feature – but equations matched)*<br>Identical equation, but poor correlation			
Inverse Variance	$$inverse\ variance = \sum_{k=1}^{N_g-1}\frac{p_{x-y}(k)}{k^2}$$	$$inverse\ variance = \sum_{i=1}^{N_g}\sum_{j=1}^{N_g}\frac{\mathbf{P}(i,j)}{\lvert i-j\rvert^2} \quad ,i \neq j$$<br>From: Aerts 2014		0.903	
Auto Correlation	$$autocorrelation = \sum_{i=1}^{N_g}\sum_{j=1}^{N_g} p(i,j)ij$$	$$f_6 = \sum_i \sum_j (ij)p(i,\ j).$$<br>From: Soh 1999<br>*Identical equation, but poor correlation*		0.366	
Cluster Prominence	$$cluster\ prominence = \sum_{i=1}^{N_g}\sum_{j=1}^{N_g}\left(i+j-\mu_x-\mu_y\right)^4 p(i,j)$$	$$f_9 = \sum_i \sum_j (i+j-\mu_x-\mu_y)^4 p(i,\ j).$$<br>From: Soh 1999		0.902	
Imc2	$$IMC\ 2 = \sqrt{1-e^{-2(HXY2-HXY)}}$$<br>$HXY = -\sum_{i=1}^{N_g}\sum_{j=1}^{N_g} p(i,j)\log_2\left(p(i,j)+\epsilon\right)$ be the entropy of $p(i,j)$<br>$HXY2 = -\sum_{i=1}^{N_g}\sum_{j=1}^{N_g} p_x(i)p_y(j)\log_2\left(p_x(i)p_y(j)+\epsilon\right)$	*12), 13) Information Measures of Correlation:*<br>$$f_{13} = (1 - \exp[-2.0(HXY2 - HXY)])^{1/2}$$<br>$$HXY = -\sum_i \sum_j p(i,j) \log(p(i,j))$$<br>$$HXY2 = -\sum_i \sum_j p_x(i)p_y(j) \log\{p_x(i)p_y(j)\}.$$<br>From: Haralick 1973<br>*Identical equation, but poor correlation*		0.475	
Imc1	$$IMC\ 1 = \frac{HXY-HXY1}{\max\{HX,HY\}}$$<br>$HXY = -\sum_{i=1}^{N_g}\sum_{j=1}^{N_g} p(i,j)\log_2\left(p(i,j)+\epsilon\right)$ be the entropy of $p(i,j)$<br>$HXY1 = -\sum_{i=1}^{N_g}\sum_{j=1}^{N_g} p(i,j)\log_2\left(p_x(i)p_y(j)+\epsilon\right)$<br>$HX = -\sum_{i=1}^{N_g} p_x(i)\log_2\left(p_x(i)+\epsilon\right)$ be the entropy of p_x<br>$HY = -\sum_{j=1}^{N_g} p_y(j)\log_2\left(p_y(j)+\epsilon\right)$ be the entropy of p_y	*12), 13) Information Measures of Correlation:*<br>$$f_{12} = \frac{HXY - HXY1}{\max\{HX,HY\}}$$<br>$$HXY = -\sum_i \sum_j p(i,j) \log(p(i,j))$$<br>$$HXY1 = -\sum_i \sum_j p(i,j) \log\{p_x(i)p_y(j)\}$$<br>From: Haralick 1973		0.88	
Cluster Tendency	$$cluster\ tendency = \sum_{i=1}^{N_g}\sum_{j=1}^{N_g}\left(i+j-\mu_x-\mu_y\right)^2 p(i,j)$$	Information dialogue references: Soh 1999<br><br>*Cluster tendency not found in this publication*	*PyRadiomics no longer calculates SumVariance as it is mathematically equivalent to ClusterTendency*	0.944	0.899

			**DEPRECATED. Sum Variance**<br>$sum\ variance = \sum_{k=2}^{2N_g} (k - SA)^2 p_{x+y}(k)$<br>Warning<br>This feature has been deprecated, as it is mathematically equal to Cluster Tendency `getClusterTendencyFeatureValue()`. See here for the proof. **Enabling this feature will result in the logging of a DeprecationWarning (does not interrupt extraction of other features), no value is calculated for this features**<br>$sum\ average = \sum_{k=2}^{2N_g} p_{x+y}(k)k$<br>*MaZda does compute sum variance*<br>$SumVarnc = \sum_{m=1}^{2^{n+1}} \left(\left(m - SumAverg \right)^2 p_{sum}(m) \right)$<br>$SumAverg = \sum_{m=1}^{2^{n+1}} \left(m\ p_{sum}(m) \right)$<br>*Difference in iteration range (1, 2^{n+1}) compared to PyRad (2, $2N_g$)*		

Supplementary Table 8: GLRLM feature equation comparison. Author comments specific to each feature are annotated by italic font.

	**PyRadiomics**	**IBEX**	**MaZda**		
General Description of Feature class & Shared equation terms	A Gray Level Run Length Matrix (GLRLM) quantifies gray level runs, which are defined as the length in number of pixels, of consecutive pixels that have the same gray level value. In a gray level run length matrix $\mathbf{P}(i,j\|\theta)$, the $(i,j)^{\text{th}}$ element describes the number of runs with gray level i and length j occur in the image (ROI) along angle θ.<br>N_g be the number of discreet intensity values in the image<br>N_r be the number of discreet run lengths in the image<br>N_p be the number of voxels in the image<br>$N_z(\theta)$ be the number of runs in the image along angle θ, which is equal to $\sum_{i=1}^{N_g}\sum_{j=1}^{N_r}\mathbf{P}(i,j\|\theta)$ and $1 \leq N_z(\theta) \leq N_p$<br>$\mathbf{P}(i,j\|\theta)$ be the run length matrix for an arbitrary direction θ<br>$p(i,j\|\theta)$ be the normalized run length matrix, defined as $p(i,j\|\theta) = \frac{\mathbf{P}(i,j\|\theta)}{N_z(\theta)}$<br>$N_g$ be the number of discreet intensit<br>N_r be the number of discreet run leng<br>N_p be the numbe<br>$N_z(\theta)$ be the nur<br>$\sum_{i=1}^{N_g}\sum_{j=1}^{N_r}\mathbf{P}(i$<br>$\mathbf{P}(i,j\|\theta)$ be the r<br>$p(i,j\|\theta)$ be the n<br><br>*No mention of angles used. Forum post refers to function "build_angles()" with mention that this is shared with GLCM, so may have similar angle behaviour?*	-Description:<br>1. This method is to compute gray-level run length matrix(GLRLM) from image inside<br>the binary mask in 2.5D in 0 and 90 degree. GLRLM summing occurence from all<br>directions is computed also. 2.5D means: First, GLRLM is computed in 2D slice by slice.<br>Then, sum the occurence of run length from all 2D image slices.<br>2. GLRLM is passed into GrayLevelRunLengthMatrix25_Feature.m to compute the related features.<br><br>-Parameters:<br>1. Direction: Define the run length direction. 0 and 90 degree are supported.<br>2. GrayLimits: Two-element vector that specifis how the grayscale values are linearly scaled into graylevels.<br>3. NumLevels: Integer specifying hte number of gray-levels to use when scaling the grayscale values.<br><br>length matrix for texture feature extraction [7]. For a given image, a run-length matrix $p(i, j)$ is defined as the number of runs with pixels of gray level i and run length j. Various texture features can then be derived from this run-length matrix.<br>let M be the number of gray levels and N<br>be the maximum run length.<br>, n_r is the total number of runs<br>and n_p is the<br>number of pixels in the image.<br>From: Tang 1997<br>*Nr equivalent to Nz(theta)*<br>*p(i,j) is equivalent to PyRad P(i,j|theta)*	The grey-level run-length matrix holds counts $p(k, l)$ of runs of pixels having the same grey level k and length l. The runs are established optionally in various directions. The feature name consist of the *Grlm* stub to indicate the grey-level run-length matrix feature extraction algorithm, then the character to indicate direction of runs: *H, V, Z, N* or *X*. The following features are computed based on the matrix.<br><br>Parameter $\Theta = 2^n - 1$ represents maximum grey level, where n is a number of bits per pixel.<br><br>$Area = \sum_{k=0}^{\Theta}\sum_{\forall l} p(k,l)$<br><br>*Area equivalent to Nz(theta)*<br><br>*p(k, l) is equivalent to PyRad: P(i,j|theta)*		

Source	PyRadiomics (2.1.0) Documentation https://pyradiomics.readthedocs.io/en/2.1.0/features.html#	IBEX software help dialogues (pop-up text descriptions available per feature, often citing an article(s) for feature details)<br><br>Galloway, Mary M. "Texture analysis using gray level run lengths." Computer graphics and image processing 4.2 (1975): 172-179.<br><br>Tang, Xiaoou. "Texture information in run-length matrices." IEEE transactions on image processing 7.11 (1998): 1602-1609.	Qmazda Documentation http://www.eletel.p.lodz.pl/pms/Programy/qmazda.pdf		
**Shared Feature Name**	**PyRadiomics**	**IBEX**	**MaZda**	**IBEX Correl.**	**MaZda Correl.**
GrayLevel Non Uniformity	$GLN = \frac{\sum_{i=1}^{N_g}\left(\sum_{j=1}^{N_r}\mathbf{P}(i,j\|\theta)\right)^2}{N_z(\theta)}$	$\mathrm{GLN} = \frac{1}{n_r}\sum_{i=1}^{M}\left(\sum_{j=1}^{N} p(i,j)\right)^2 = \frac{1}{n_r}\sum_{i=1}^{M} p_g(i)^2.$<br>From: Tang 1998	$GLevNonUni = \frac{1}{Area}\sum_{k=0}^{\Theta}\left(\sum_{\forall l} p(k,l)\right)^2$	-0.053	0.942
RunLength Non Uniformity	$RLN = \frac{\sum_{j=1}^{N_r}\left(\sum_{i=1}^{N_g}\mathbf{P}(i,j\|\theta)\right)^2}{N_z(\theta)}$	$\mathrm{RLN} = \frac{1}{n_r}\sum_{j=1}^{N}\left(\sum_{i=1}^{M} p(i,j)\right)^2 = \frac{1}{n_r}\sum_{j=1}^{N} p_r(i)^2.$<br>From: Tang 1998	$RLNonUni = \frac{1}{Area}\sum_{\forall l}\left(\sum_{k=0}^{\Theta} p(k,l)\right)^2$	-0.038	0.848
GrayLevel Non Uniformity Normalized	$GLNN = \frac{\sum_{i=1}^{N_g}\left(\sum_{j=1}^{N_r}\mathbf{P}(i,j\|\theta)\right)^2}{N_z(\theta)^2}$		$MGLevNonUni = \frac{1}{Area^2}\sum_{k=0}^{\Theta}\left(\sum_{\forall l} p(k,l)\right)^2$<br>*Names similar, equations same, correlation poor.*		0.657
RunLength Non Uniformity Normalized	$RLNN = \frac{\sum_{j=1}^{N_r}\left(\sum_{i=1}^{N_g}\mathbf{P}(i,j\|\theta)\right)^2}{N_z(\theta)^2}$		$MRLNonUni = \frac{1}{Area^2}\sum_{\forall l}\left(\sum_{k=0}^{\Theta} p(k,l)\right)^2$<br>*Names similar, equations same, correlation moderate.*		0.892

LongRun Emphasis	$LRE = \frac{\sum_{i=1}^{N_g} \sum_{j=1}^{N_r} \mathbf{P}(i,j\|\theta) j^2}{N_z(\theta)}$	$\mathrm{LRE} = \frac{1}{n_r} \sum_{i=1}^{M} \sum_{j=1}^{N} p(i,j) \cdot j^2 = \frac{1}{n_r} \sum_{j=1}^{N} p_r(j) \cdot j^2.$ From: Tang 1998	$LongREmph = \frac{1}{Area} \sum_{k=0}^{\Theta} \sum_{\forall l} l^2 p(k,l)$	-0.194	0.906
ShortRun Emphasis	$SRE = \frac{\sum_{i=1}^{N_g} \sum_{j=1}^{N_r} \frac{\mathbf{P}(i,j\|\theta)}{j^2}}{N_z(\theta)}$	$\mathrm{SRE} = \frac{1}{n_r} \sum_{i=1}^{M} \sum_{j=1}^{N} \frac{p(i,j)}{j^2} = \frac{1}{n_r} \sum_{j=1}^{N} \frac{p_r(j)}{j^2}.$ From: Tang 1998	$ShrtREmph = \frac{1}{Area} \sum_{k=0}^{\Theta} \sum_{\forall l} \frac{p(k,l)}{l^2}$	-0.046	0.893
Run Percentage	$RP = \frac{N_z(\theta)}{N_p}$	$\mathrm{RP} = \frac{n_r}{n_p}.$ From: Tang 1997 Same name, same equation, poor correlation	$Fraction = \frac{\sum_{k=0}^{\Theta} \sum_{\forall l} p(k,l)}{\sum_{k=0}^{\Theta} \sum_{\forall l} l\, p(k,l)}$ *Similar name, Different denominator. Good correlation*	-0.13	0.926
ShortRun LowGrayLvl Emp	$SRLGLE = \frac{\sum_{i=1}^{N_g} \sum_{j=1}^{N_r} \frac{\mathbf{P}(i,j\|\theta)}{i^2 j^2}}{N_z(\theta)}$	$\mathrm{SRLGE} = \frac{1}{n_r} \sum_{i=1}^{M} \sum_{j=1}^{N} \frac{p(i,j)}{i^2 \cdot j^2}.$ From: Tang 1998		0.175	
LowGrayLevel RunEmp	$LGLRE = \frac{\sum_{i=1}^{N_g} \sum_{j=1}^{N_r} \frac{\mathbf{P}(i,j\|\theta)}{i^2}}{N_z(\theta)}$	$\mathrm{LGRE} = \frac{1}{n_r} \sum_{i=1}^{M} \sum_{j=1}^{N} \frac{p(i,j)}{i^2} = \frac{1}{n_r} \sum_{i=1}^{M} \frac{p_g(i)}{i^2}.$ From: Tang 1998		0.213	

ShortRun HighGrayLvl Emp	$SRHGLE = \frac{\sum_{i=1}^{N_g}\sum_{j=1}^{N_r}\frac{\mathbf{P}(i,j\|\theta)i^2}{j^2}}{N_z(\theta)}$	$\text{SRHGE} = \frac{1}{n_r}\sum_{i=1}^{M}\sum_{j=1}^{N}\frac{p(i,j)\cdot i^2}{j^2}.$<br>From: Tang 1998		0.371	
LongRun HighGrayLvl Emp	$LRHGLRE = \frac{\sum_{i=1}^{N_g}\sum_{j=1}^{N_r}\mathbf{P}(i,j\|\theta)i^2j^2}{N_z(\theta)}$	4) *Long Run High Gray-Level Emphasis (LRHGE):*<br>$\text{LRLGE} = \frac{1}{n_r}\sum_{i=1}^{M}\sum_{j=1}^{N} p(i,j)\cdot i^2\cdot j^2.$<br>*Typo in paper (LRLGRE).* From Tang 1998		0.22	
LongRun LowGrayLvl Emp	$LRLGLRE = \frac{\sum_{i=1}^{N_g}\sum_{j=1}^{N_r}\frac{\mathbf{P}(i,j\|\theta)j^2}{i^2}}{N_z(\theta)}$	$\text{LRLGE} = \frac{1}{n_r}\sum_{i=1}^{M}\sum_{j=1}^{N}\frac{p(i,j)\cdot j^2}{i^2}.$<br>From: Tang 1997		0.395	
HighGrayLvl RunEmp	$HGLRE = \frac{\sum_{i=1}^{N_g}\sum_{j=1}^{N_r}\mathbf{P}(i,j\|\theta)i^2}{N_z(\theta)}$	$\text{HGRE} = \frac{1}{n_r}\sum_{i=1}^{M}\sum_{j=1}^{N} p(i,j)\cdot i^2 = \frac{1}{n_r}\sum_{i=1}^{M} p_g(i)\cdot i^2.$<br>From: Tang 1997		0.366	

Supplementary Table 9: NGTDM feature equation comparison. Author comments specific to each feature are annotated by italic font

	**PyRadiomics**	**IBEX**	**MaZda**		
General Description of Feature class & Shared equation terms	A Neighbouring Gray Tone Difference Matrix quantifies the difference between a gray value and the average gray value of its neighbours within distance δ. The sum of absolute differences for gray level i is stored in the matrix. Let $\mathbf{X}_{gl}$ be a set of segmented voxels and $x_{gl}(j_x,j_y,j_z) \in \mathbf{X}_{gl}$ be the gray level of a voxel at postion (j_x,j_y,j_z), then the average gray level of the neigbourhood is:<br>$\bar{A}_i = \bar{A}(j_x,j_y,j_z) = \frac{1}{W}\sum_{k_x=-\delta}^{\delta}\sum_{k_y=-\delta}^{\delta}\sum_{k_z=-\delta}^{\delta} x_{gl}(j_x+k_x, j_y+k_y, j_z+k_z),$<br>where $(k_x,k_y,k_z) \neq (0,0,0)$ and $x_{gl}(j_x+k_x, j_y+k_y, j_z+k_z) \in \mathbf{X}_{gl}$<br>Here, W is the number of voxels in the neighbourhood that are also in $\mathbf{X}_{gl}$.<br><br>n_i be the number of voxels in X_{gl} with gray level i<br>$N_{v,p}$ be the total number of voxels in X_{gl} and equal to $\sum n_i$ (i.e. the number of voxels with a valid region; at least 1 neighbor). $N_{v,p} \leq N_p$, where N_p is the total number of voxels in the ROI.<br>p_i be the gray level probability and equal to n_i/N_v<br>$s_i = \begin{cases} \sum^{n_i} \lvert i-\bar{A}_i \rvert & \text{for} \quad n_i \neq 0 \\ 0 & \text{for} \quad n_i = 0 \end{cases}$ be the sum of absolute differences for gray level i<br>N_g be the number of discreet gray levels<br>$N_{g,p}$ be the number of gray levels where $p_i \neq 0$<br>The following class specific settings are possible:<br>• distances [[1]]: List of integers. This specifies the distances between the center voxel and the neighbor, for which angles should be generated.<br>References<br>• Amadasun M, King R; Textural features corresponding to textural properties; Systems, Man and Cybernetics, IEEE Transactions on 19:1264-1274 (1989). doi: 10.1109/21.44046<br>*N_v undefined in p_i definition, potentially referring to $N_{v,p}$?*	-Description:<br>1. This method is to compute neighborhood intensity difference matrix(NIDM) from image inside the binary mask. The neighborhood is in 2D. Intensity difference is computed in 2D neighborhood. All the feature calculation is done the same as NeighborIntensityDifference3 does.<br>2. NIDM is passed into NeighborIntensityDifference25_Feature.m to compute the related features.<br><br>-Parameters:<br>1. NHood: The neighborhood matrix size in X dimension.<br>2. NHoodSym: 1==neighborhood matrix size in Y are calculated to best match neighborhood physical length in X dimension.<br>0==neighborhood matrx size are same in X and Y dimensions.<br>3. IncludeEdge: Include edge pixels for analysis (1) or not (0).<br>4. AdaptLimitLevel: If AdaptLimitLevel=1, ignore parameters RangeMin, RangeMax, and NBins. Range set to the minimum(MinValue) and maximum(MaxValue) of the masked image. NBins is length(MinValue:MaxValue). If RoundtoNearest is used for preprocessing, Nbins = length(MinValue:Value:MaxValue) where value is from the parameter for RoundtoNearest.<br>5. RangeMin: Minimum intensity value for analysis.<br>6. RangeMax: Maximum intensity value for analysis.<br>7. NBins: The number of bins.<br>RangeMin, RangeMax, NBins are used to reduce the number of interested intensity level.<br><br>*For all features there main reference is given*<br>This is a column matrix formed as follows.<br>Let $f(k,l)$ be the gray tone of any pixel at (k,l) having gray tone value i. Then we find the average gray-tone over a neighborhood centered at, but excluding (k,l)<br>$\bar{A}_i = \bar{A}(k,l) = \frac{1}{W-1}\left[\sum_{m=-d}^{d}\sum_{n=-d}^{d} f(k+m, l+n)\right]$ $(m,n) \neq (0,0)$, (1)<br>where d specifies the neighborhood size and $W = (2d+1)^2$.<br>Then the ith entry in the NGTDM is<br>$s(i) = \sum \lvert i - \bar{A}_i \rvert$, for $i \in N_i$ if $N_i \neq 0$,<br>$= 0$, otherwise (2)<br>where $\{N_i\}$ is the set of all pixels having gray tone i (except in the peripheral regions of width d).<br>For an $N \times N$ image, p_i is the probabiity of occurrence of gray-tone value i, and is given by<br>$p_i = N_i/n^2$, where $n = N - 2d$.<br>*Hard to assess if p_i is mathematically equivalent to Pyradiomics p_i. Additionally, is the common term n^2 equivalent to PyRadiomics $N_{v,p}$?*	No NGTDM feature option.		

Source	PyRadiomics (2.1.0) Documentation<br>https://pyradiomics.readthedocs.io/en/2.1.0/features.html#	IBEX software help dialogues (pop-up text descriptions available per feature, often citing an article(s) for feature details)<br>Amadasun, Moses, and Robert King. "Textural features corresponding to textural properties." IEEE Transactions on systems, man, and Cybernetics 19.5 (1989): 1264-1274.	Qmazda Documentation<br>http://www.eletel.p.lodz.pl/pms/Programy/qmazda.pdf		
**Shared Feature Name**	**PyRadiomics**	**IBEX**	**MaZda**	**IBEX Correl.**	**MaZda Correl.**
Coarseness	$Coarseness = \frac{1}{\sum_{i=1}^{N_g} p_i s_i}$<br>N.B. $\sum_{i=1}^{N_g} p_i s_i$ potentially evaluates to 0 (in case of a completely homogeneous image). If this is the case, an arbitrary value of 10^6 is returned.	$f_{\cos} = \left[\epsilon + \sum_{i=0}^{G_h} p_i s(i)\right]^{-1}$ (3)<br>where G_h is the highest gray-tone value present in the image and ϵ is a small number to prevent $f_{\cos}$ becoming infinite.<br>*Different divide 0 error handling, poor correlation*		-0.099	
Complexity	$Complexity = \frac{1}{N_{v,p}} \sum_{i=1}^{N_g}\sum_{j=1}^{N_g} \|i-j\| \frac{p_i s_i + p_j s_j}{p_i + p_j}$, where $p_i \neq 0, p_j \neq 0$	$f_{com} = \sum_{i=0}^{G_h}\sum_{j=0}^{G_h} \{(\|i-j\|)/(n^2(p_i+p_j))\}\{p_i s(i) + p_j s(j)\},$<br>$p_i \neq 0, p_j \neq 0.$ (6)<br>*Is n^2 equivalent to $N_{v,p}$, good correlation*		0.931	
Strength	$Strength = \frac{\sum_{i=1}^{N_g}\sum_{j=1}^{N_g}(p_i+p_j)(i-j)^2}{\sum_{i=1}^{N_g} s_i}$, where $p_i \neq 0, p_j \neq 0$<br>N.B. $\sum_{i=1}^{N_g} s_i$ potentially evaluates to 0 (in case of a completely homogeneous image). If this is the case, 0 is returned.	$f_{str} = \left[\sum_{i=0}^{G_h}\sum_{j=0}^{G_h}(p_i+p_j)(i-j)^2\right] / \left[\epsilon + \sum_{i=0}^{G_h} s(i)\right],$<br>$p_i \neq 0, p_j \neq 0.$ (7)<br>*Different divide 0 error handling, moderate correlation*		0.861	

Contrast	$Contrast = \left(\frac{1}{N_{g,p}(N_{g,p}-1)}\sum_{i=1}^{N_g}\sum_{j=1}^{N_g} p_i p_j (i-j)^2\right)\left(\frac{1}{N_{v,p}}\sum_{i=1}^{N_g} s_i\right)$, where $p_i \neq 0, p_j \neq 0$<br>N.B. In case of a completely homogeneous image, $N_{g,p} = 1$, which would result in a division by 0. In this case, an arbitray value of 0 is returned.	$f_{con} = \left[\frac{1}{N_g(N_g-1)}\sum_{i=0}^{G_h}\sum_{j=0}^{G_h} p_i p_j (i-j)^2\right]\left[\frac{1}{n^2}\sum_{i=0}^{G_h} s(i)\right]$ (4)<br>where N_g is the total number of different gray levels present in the image.<br>$N_g = \sum_{i=0}^{G_h} Q_i$, where $Q_i = \begin{cases} 1, & \text{if } p_i \neq 0 \\ 0, & \text{otherwise.} \end{cases}$<br>*Is n^2 equivalent to $N_{v,p}$, moderate correlation*		0.891	
Busyness	$Busyness = \frac{\sum_{i=1}^{N_g} p_i s_i}{\sum_{i=1}^{N_g}\sum_{j=1}^{N_g} \lvert ip_i - jp_j \rvert}$, where $p_i \neq 0, p_j \neq 0$<br>N.B. if $N_{g,p} = 1$, then $busyness = \frac{0}{0}$. If this is the case, 0 is returned, as it concerns a fully homogeneous region.	$f_{bus} = \left[\sum_{i=0}^{G_h} p_i s(i)\right] \Big/ \left[\sum_{i=0}^{G_h}\sum_{j=0}^{G_h} ip_i - jp_j\right]$, $p_i \neq 0, p_j \neq 0.$<br>*Similar equation. Absolute operator in denominator missing*		-0.049	
